# Foliar Pine Pathogens From Different Kingdoms Share Defence‐Eliciting Effector Proteins

**DOI:** 10.1111/mpp.70065

**Published:** 2025-03-02

**Authors:** Mariana Tarallo, Carl H. Mesarich, Rebecca L. McDougal, Rosie E. Bradshaw

**Affiliations:** ^1^ School of Food Technology and Natural Sciences Massey University Palmerston North New Zealand; ^2^ Bioprotection Aotearoa Lincoln New Zealand; ^3^ School of Agriculture and Environment Massey University Palmerston North New Zealand; ^4^ Scion (The New Zealand Forest Research Institute Ltd.) Rotorua New Zealand

**Keywords:** cell death‐eliciting effectors, cross‐kingdom effectors, gene disruption, pine pathogens, plant–pathogen interactions, protein tertiary structures

## Abstract

Dothistroma needle blight, Cyclaneusma needle blight and red needle cast are devastating foliar pine diseases caused by the fungi *Dothistroma septosporum* and *Cyclaneusma minus* and the oomycete *Phytophthora pluvialis*, respectively. These pathogens colonise the host apoplast, secreting effector proteins to promote infection and disease. If these effectors are recognised by corresponding host resistance proteins, they activate the plant immune system to stop pathogen growth. We aimed to identify and characterise effectors that are common to all three pathogens. Using *D. septosporum* as a starting point, three candidate effectors (CEs) were investigated: Ds69335 (a CAP protein) and Ds131885, both of which have sequence and structural similarity to CEs of *C. minus* and *P. pluvialis* , and Ds74283, which adopts a β‐trefoil fold and has structural rather than sequence similarity to CEs from all three pathogens. Notably, of the CEs investigated, Ds74283 and Ds131885, as well as their homologues from *C. minus* and *P. pluvialis* , elicited chlorosis or cell death in *Nicotiana* species, with Ds131885 and its homologues also triggering cell death in *Pinus radiata* . In line with these observed responses being related to activation of the plant immune system, the chlorosis triggered by Ds131885 and its homologues was compromised in a *Nicotiana benthamiana* mutant lacking the extracellular immune system co‐receptor, SOBIR1. Such cross‐kingdom, plant immune system‐activating effectors, whether similar in sequence or structure, might ultimately enable the selection or engineering of durable, broad‐spectrum resistance against foliar pine pathogens.

## Graphical abstract

This study identified shared plant immune‐activating effectors between pine pathogens *Dothistroma septosporum*, *Cyclaneusma minus* and *Phytophthora pluvialis*, which could aid in developing broad‐spectrum disease resistance.

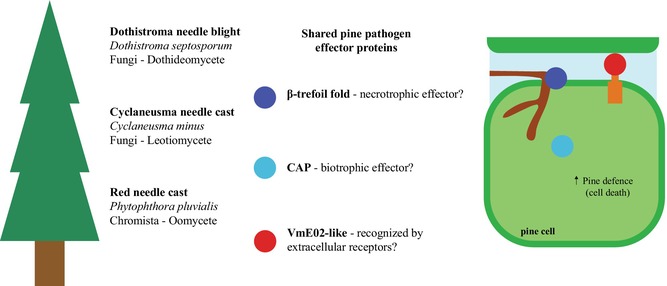

## 1 Introduction

To protect themselves against infection and disease, plants have an efficient immune system that is built on the recognition of non‐self (Bentham et al. 2020). This immune system has an extracellular (apoplastic) component in which, following pathogen entry, broadly conserved molecules, termed pathogen‐associated molecular patterns (PAMPs), are detected by cell surface‐localised immune receptors of the pattern recognition receptor (PRR) class. This recognition often involves association with immune co‐receptors such as BAK1 and SOBIR1 and triggers a signal transduction cascade that activates defence responses to prevent or slow further pathogen ingress (Hogenhout et al. 2009; Huang et al. 2021). The amplification of these signals occurs through the actions of regulatory hormones such as salicylic acid (SA), jasmonic acid (JA) and ethylene (ET), resulting in the activation of transcription factors, including those of the WRKY superfamily, defence genes, pathogenesis‐related (PR) proteins, phytoalexins, lignification of tissues and deposition of callose and other cell wall reinforcements (Blázquez et al. 2020). In an attempt to suppress these defence responses, pathogens deliver virulence and pathogenicity factors, termed effectors, into and around plant cells (He et al. 2020; Rocafort et al. 2020); these effectors are called intracellular and extracellular effectors, respectively.

Fungal effectors, which are typically small proteins, do not usually possess conserved motifs or domains and can be species‐specific (Lo Presti et al. 2015; Rocafort et al. 2020). There are, however, some ‘core effectors’ that are highly conserved between fungal taxa. These effectors provide a core function that is likely important to the virulence or pathogenicity of the fungi that produce them (Chepsergon et al. 2021). In some cases, effectors are shared across kingdoms, with core effectors from fungi, oomycetes and even bacteria sharing conserved domains. For example, the necrosis and ethylene‐inducing peptide 1‐like proteins and the glycoside hydrolase 12 protein XEG1 are broadly distributed in oomycetes and fungi, and both types elicit host defence responses (Fellbrich et al. 2002; Ma et al. 2015). Despite this fact, very little is known about cross‐kingdom effectors and their potential roles in pathogen–host interactions, particularly those involving gymnosperms. Identifying cross‐kingdom or ‘shared’ effectors is important, as disease resistance is more likely to be durable if it is based on core effectors that are important for a pathogen's ability to cause disease.

The fungal pathogens *Dothistroma septosporum* and *Cyclaneusma minus*, and the oomycete pathogen *Phytophthora pluvialis*, each have serious impacts on *Pinus radiata* and other pine species around the world, causing Dothistroma needle blight, Cyclaneusma needle cast and red needle cast, respectively (Bednářová et al. 2013; Drenkhan et al. 2016; Reeser et al. 2013). Despite being distantly related, all three pathogens colonise the apoplast, where they secrete effector proteins to promote infection and disease (Dick et al. 2014; Kabir et al. 2015). Notably, however, plants also have an intracellular component to their immune system, in which intracellular effectors can be recognised by corresponding resistance (R) proteins of the nucleotide‐binding domain and leucine‐rich repeat class (Cook et al. 2015). This recognition, or the recognition of extracellular effectors by PRRs at the cell surface which, in this case, also act as R proteins, triggers a signal transduction cascade that activates defence responses to stop pathogen growth. Here, the major defence response is typically a localised cell death reaction called the hypersensitive response (HR), with the recognised effector called an avirulence factor (Win et al. 2012). Finding common virulence or avirulence factors can provide important insights into how foliar pine pathogens interact with their hosts to cause disease and, ultimately, how pines recognise and defend themselves against these pathogens.

Due to the constant ‘arms race’ between plants and pathogens, effector genes usually evolve more quickly than the rest of a pathogen's genome. This is reflected in duplication events, loss and gain of genes, and lack of primary sequence identity between effector proteins from different pathogens that nonetheless in some cases still have similar tertiary structures and share common host targets (Fouché et al. 2018). Effector proteins can share similar structural folds despite no or little sequence identity, which suggests divergent evolution and/or selection has occurred to maintain an important structure (Andrie et al. 2008; de Guillen et al. 2015; Franceschetti et al. 2017; Seong and Krasileva 2021, 2023). Shared folds between sequence‐unrelated effector proteins suggest their functional importance and demonstrate how tertiary structure predictions can contribute to better understanding their roles in phytopathogenic interactions. For example, MAX effectors from *Magnaporthe oryzae* share a common fold that could not be detected by sequence similarity (Seong and Krasileva 2021). Indeed, many pathogen effectors can be grouped into large, structurally similar families (Derbyshire and Raffaele 2023; Rocafort, Bowen, et al. 2022; Seong and Krasileva 2023; Yu et al. 2024). Therefore, identifying core effectors, either by sequence or structural similarity, is important due to their possible essential roles in virulence. The subsequent identification of plant R proteins that can recognise such core effectors could be beneficial for breeding and selection programmes. The recognition by R proteins of a conserved group of effector molecules can confer both durable and broad‐spectrum resistance against microbes sharing those conserved effectors (Dalio et al. 2017); this would be particularly beneficial for trees, such as pines, that have long life cycles.

In a previous study, we identified candidate effector (CE) proteins from *D. septosporum*, some of which triggered cell death responses in non‐host *Nicotiana* plants (Hunziker et al. 2021). Moreover, some of these proteins, also present in *C. minus* , were predicted to adopt a common β‐trefoil fold (Tarallo et al. 2022, 2024). The aim of this work was to identify and characterise homologues of *D. septosporum* CE proteins in *C. minus* and *P. pluvialis* . This work provides unique insights into cross‐kingdom effectors of gymnosperm pathogens that may have implications for resistance breeding or engineering in pines.

## 2 Results

### 2.1 Identification of Common Candidate Effector Proteins From *Dothistroma septosporum*, *Cyclaneusma minus* and *Phytophthora pluvialis*

Thirty in planta‐expressed CE genes that are each predicted to encode a secreted protein were previously identified from the *D. septosporum* NZE10 genome (Bradshaw et al. 2016; Hunziker et al. 2021). To assess which of these 30 CEs are potentially common between foliar pine pathogens, each was screened against the genomes and predicted proteomes of *C. minus* NZFS809 and *P. pluvialis* NZFS3000 using BLAST. Based on these searches, 10 of the 30 CEs had one or more significant BLAST hits in *C. minus* (Table S1). Of these 10, two also had one significant BLAST hit in *P. pluvialis* (Table S1), suggesting that they are ‘cross‐kingdom’ CEs.

The first set of cross‐kingdom CE proteins that are common between the three pine needle pathogens consisted of Ds69335 (*D. septosporum*), Cm8840 ( *C. minus* ) and Pp7927 ( *P. pluvialis* ), with Ds69335 previously identified as belonging to the ‘cysteine‐rich secretory proteins, antigen 5, and pathogenesis‐related 1 proteins’ (CAP) superfamily (Hunziker et al. 2021) (Table S1). All three proteins were predicted to have an intrinsically disordered region (IDR), with Ds69335 and Cm8840 having an N‐terminal IDR and Pp7927 having a C‐terminal IDR approximately 160 amino acids longer than those of the other proteins (Table S2).

The second set of cross‐kingdom CE proteins shared between the three pine needle pathogens comprised Ds131885 (*D. septosporum*), Cm2721 ( *C. minus* ) and Pp10632 ( *P. pluvialis* ), with full‐length pairwise amino acid identities of more than 64% observed between each pair (Table S1). Ds131885 is an orthologue of VmE02, a cross‐kingdom PAMP from the apple‐pathogenic fungus, *Valsa mali* (Hunziker et al. 2021; Nie et al. 2019). All three proteins were predicted to possess a signal peptide, contain no characterised functional domains, and have at least 10 conserved cysteine residues (Table S2).

### 2.2 *Dothistroma septosporum*, *Cyclaneusma minus* and *Phytophthora pluvialis* Secrete CAP Proteins With a Potential Role in Virulence

**FIGURE 1:**
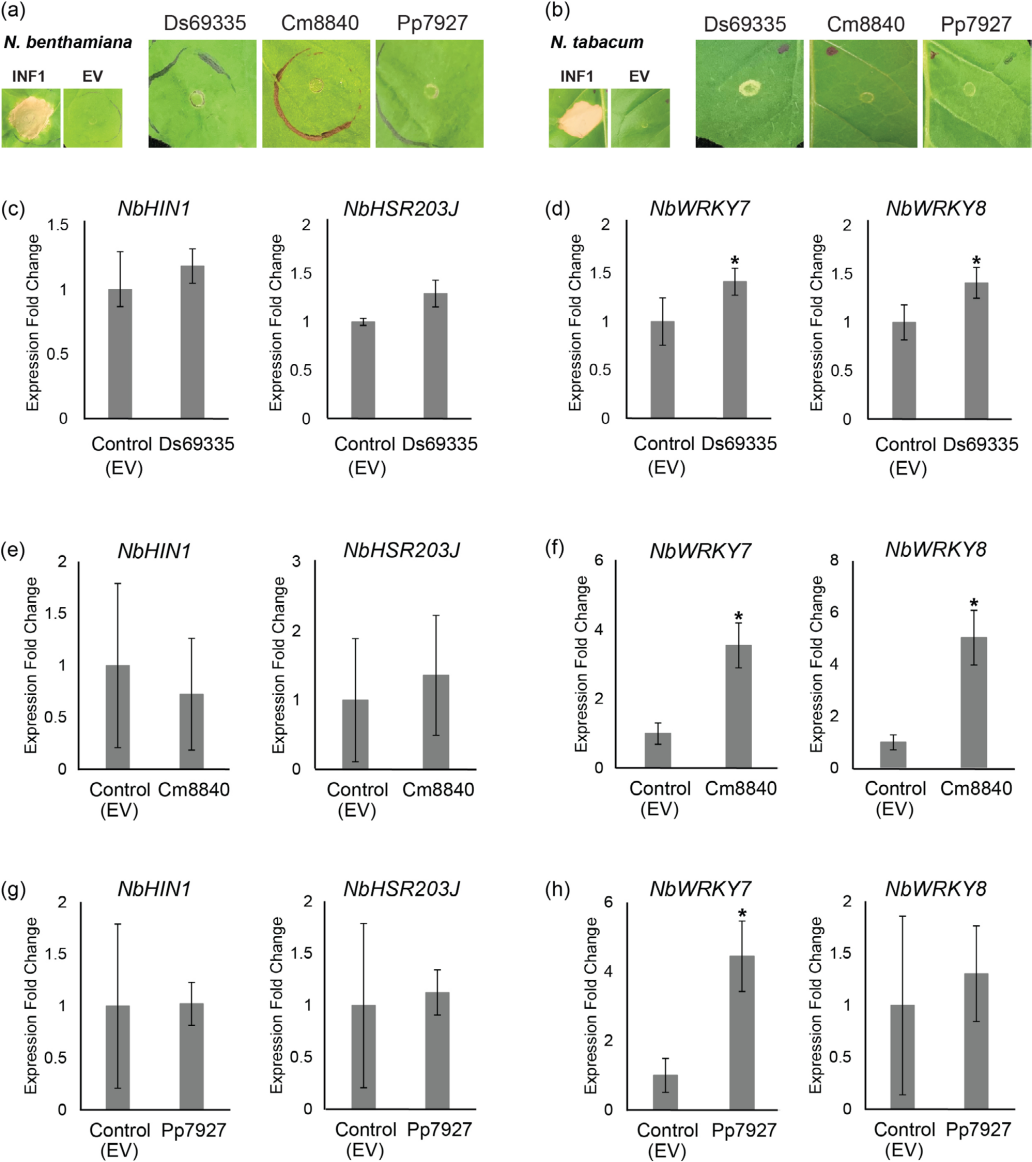
*Dothistroma septosporum*, *Cyclaneusma minus* and *Phytophthora pluvialis* are predicted to secrete proteins belonging to the CAP superfamily. The most closely related CAP proteins between *D. septosporum* (Ds69335), *C. minus* (Cm8840) and *P. pluvialis* (Pp7927) were transiently expressed in *Nicotiana benthamiana* (a) and *Nicotiana tabacum* (b). Transient expression of Ds69335 was repeated according to Hunziker et al. (2021). INF1, *Phytophthora infestans* elicitin positive cell death control; EV, empty vector negative no cell death control. Expression of (c, e and g) hypersensitive‐response‐specific marker genes (*NbHIN1* and *NbHSR203J*) and (d, f and h) pathogen‐associated molecular pattern‐triggered immunity marker genes (*NbWRKY7* and *WRKY8*) in *N. benthamiana*. *Ds69335*, *Cm8840*, *Pp7927* and pICH86988 EV control were transiently expressed in *N. benthamiana* and leaves sampled after 48 h. Means and standard errors of normalised expression values were calculated from at least three biological replicates. *p < 0.05.

Ds69335 is strongly upregulated at the early (biotrophic) stage of pine infection by *D. septosporum* (Bradshaw et al. 2016) and does not elicit any visible cell death response in *Nicotiana benthamiana* and *Nicotiana tabacum* (Hunziker et al. 2021) (Figure S1a). With the latter point in mind, we assessed whether the homologues from *C. minus* (Cm8840) and *P. pluvialis* (Pp7927) cou trigger chlorosis or cell death in *N. benthamiana* and/or *N. tabacum* . As previously observed for Ds69335, the Cm8840 and Pp7927 proteins did not trigger chlorosis or cell death in either of the *Nicotiana* species tested (Figure 1a,b).

To determine if transient expression of Ds69335, Cm8840 and Pp7927 in *N. benthamiana* triggered plant immune responses that were not visible with the naked eye, the expression of defence marker genes was analysed by reverse transcription‐quantitative PCR (RT‐qPCR). For *N. benthamiana* plants expressing Ds69335, Cm8840 or Pp7927, neither of the two HR‐specific marker genes tested, *NbHIN1* and *NbHSR203J* (Pontier et al. 1994; Takahashi et al. 2004), were transcriptionally upregulated 48 h after inoculation (hai) (Figure 1c,e,g). Because these proteins do not trigger visible chlorosis or cell death in *N. benthamiana*, these HR‐specific markers were not expected to be upregulated. Similarly, there was no alteration in the expression of genes related to hormone signalling pathways (SA, JA and ET) (Figure S2). However, at 48 hai, two PAMP‐triggered immunity (PTI) markers, *NbWRKY7* and *NbWRKY8* (McLellan et al. 2013), were transcriptionally upregulated in *N. benthamiana* plants expressing Ds69335, Cm8840 or Pp7927 (Figure 1d,f,h).

**FIGURE 2:**
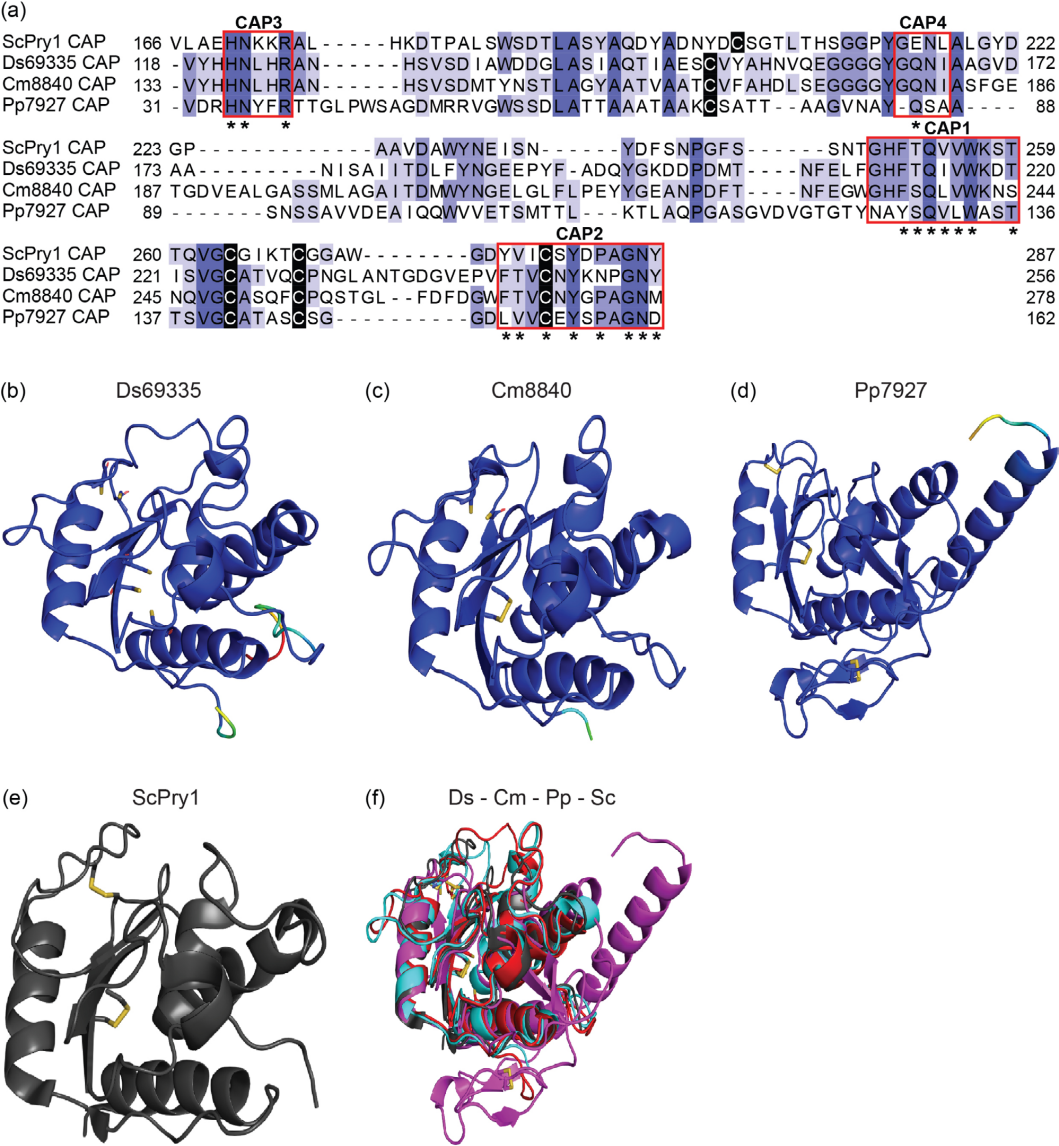
Ds69335, Cm8840 and Pp7927 are structurally similar to Pry1 from *Saccharomyces cerevisiae* . (a) Amino acid alignment of the CAP domains of Ds69335, Cm8840 and Pp7927 with the CAP domain of ScPry1 from *S. cerevisiae* (Darwiche et al. 2016). Cysteine residues are highlighted in black. Asterisks (*) refer to conserved motifs CAP1–4 present in other CAP proteins (Han et al. 2023). Predicted tertiary structures of Ds69335 (b), Cm8840 (c) and Pp7927 (d). For Ds69335, AlphaFold2 predicted a tertiary structure with a pLDDT score of 74.9 and a predicted TM‐score of 0.69, 74.1 and 0.65 for Cm8840 and 68.1 and 0.5 for Pp7927. Disulphide bonds and/or cysteine residues are shown as yellow sticks. Structures are shown without their predicted intrinsically disordered region (see Figure S3 for full structures). (e) Characterised structure of Pry1 from *S. cerevisiae* (PBD ID: 5jys) (Darwiche et al. 2016). (f) Alignment of predicted Ds69335 (red), Cm8840 (cyan blue), Pp7927 (magenta) and Pry1 (grey).

Like Ds69335, both Cm8840 and Pp7927 contain a conserved domain of the CAP superfamily (Figure 2a). Most plant and fungal CAP proteins have four sequence motifs (CAP1–4) (Han et al. 2023). All four motifs are present in the three CE proteins; however, the motifs of Pp7927 are more divergent from the consensus sequence (Han et al. 2023) than those in Ds69335 and Cm8840 (Figure 2a). Tertiary structure predictions of the three CAP domain‐containing CE proteins revealed their strong structural similarity to pathogen‐related yeast 1 (Pry1) (RCSB PDB ID 5jys), a sterol and lipid‐binding CAP protein from the yeast *Saccharomyces cerevisiae* (Darwiche et al. 2016) (Figures 2f and S3). Figure 2 shows the predicted tertiary structures of Ds69335, Cm8840 and Pp7927 without the putative IDRs. The complete predicted tertiary structures of these proteins, including IDRs, are in Figure S3.

To determine whether Ds69335 contributes to the virulence of *D. septosporum* during infection of *P. radiata* , we generated *Ds69335*‐disrupted mutants using CRISPR/Cas9, as well as a version of a disrupted strain in which a wild‐type (WT) copy of the *Ds69335* gene had been reintroduced (complementation strain). Mutants with a disruption in *Ds69335* were confirmed by PCR and Southern blot hybridization (Figure S4), while the complementation strain carrying a single WT copy of *Ds69335* was confirmed by qPCR (Table S3). In all cases, the selected mutant and complementation strains exhibited normal growth and sporulation rates during growth in culture when compared to the WT fungus (Table S4).

The virulence assay showed that one of the *Ds69335*‐disrupted mutants of *D. septosporum* produced significantly less fungal biomass during infection of *P. radiata* , per mg/dry weight (DW) of infected plant tissue, when compared to the WT fungus. A second independent *Ds69335*‐disrupted mutant showed no significant difference to the WT fungus overall (Table 1), but closer inspection revealed three of the four replicate inoculations of pine produced less fungal biomass than the WT, whilst the other replicate was an outlier (File S1). However, unexpectedly, the complementation strain also produced significantly less fungal biomass than the WT fungus (Table 1). Because the complementation strain was not restored to the same level of virulence as the WT fungus, no clear conclusion could be drawn regarding the contribution of Ds69335 to the virulence of *D. septosporum* during infection of *P. radiata* . Details of these results are shown in File S1.

**TABLE 1:** Relative quantification of *Dothistroma septosporum* candidate effector gene disruption and complementation strain biomass in *Pinus radiata* .

*DsCE* gene^a^	Strain name^b^	Fungal DNA (rel quant) (ng/mg DW)^c^	*p* (*t* test) WT/Co^d^
**Set A**			
	WT	7.1 ± 0.6	—
*Ds69335*	*Ds69335* T2‐D	3.6 ± 1.6	0.025*/0.23
	*Ds69335* T40‐D	3.0 ± 2.7	0.078/0.58
	*Ds69335* T2‐Co	1.9 ± 1.5	0.006*/—
*Ds74283*	*Ds74283* T3‐D	2.4 ± 1.5	0.007*/0.51
	*Ds74283* T56‐D	2.7 ± 1.0	0.003*/0.24
	*Ds74283* T3‐Co	1.6 ± 1.0	0.001*/—
*Ds131885*	*Ds131885* T9‐Co	1.3 ± 1.9	0.008*/—
**Set B**			
	WT	2.0 ± 0.5	—
*Ds131885*	*Ds131885* T9‐D (L)	3.5 ± 1.6	0.30/0.24
	*Ds131885* T9‐D (M)	0.7 ± 0.2	0.03*/0.65
	*Ds131885* T47‐D	3.3 ± 1.0	0.18/0.22

^a^ Joint Genome Institute (JGI) protein ID corresponding to the *Dothistroma septosporum* NZE10 (*Ds*) candidate effector (*CE*) gene of interest that was disrupted.

^b^ *D. septosporum* strains with a targeted gene disruption are indicated by “D”; complementation strains are indicated by “Co”. WT: wild‐type. Low (L) and moderate (M) concentration of spores used for inoculation of *Pinus radiata* seedlings according to File File S1.

^c^ Fungal biomass (ng) relative to pine and normalised to dry weight (DW) of infected needle tissue (mean ± SD). Full data are shown in Tarallo et al. (2022).

^d^ *p*‐value, using Student's *t* test, between WT and each transformant (first value) and between the disruption strain and its respective complementation strain (second value). The asterisk * indicates a significant difference (*p* < 0.05).

### 2.3 A Predicted PAMP Is Conserved Between the Three Pine Needle Pathogens

**FIGURE 3:**
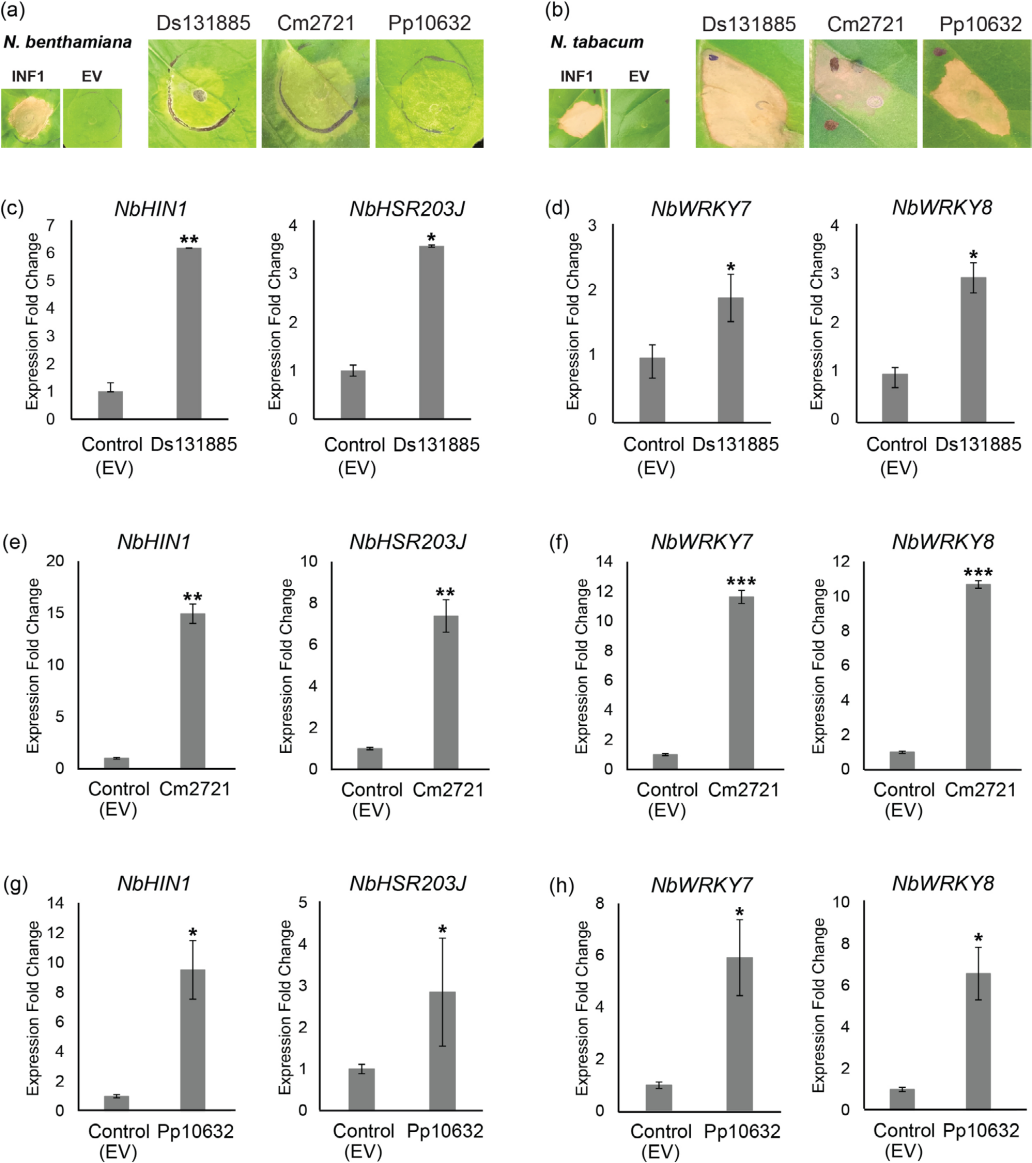
*Dothistroma septosporum*, *Cyclaneusma minus* and *Phytophthora pluvialis* each secrete a protein similar to a characterised cross‐kingdom pathogen‐associated molecular pattern (PAMP) called VmE02 from *Valsa mali*. Homologues between *D. septosporum* (Ds131885), *C. minus* (Cm2721) and *P. pluvialis* (Pp10632) were transiently expressed in *Nicotiana benthamiana* (a) and *Nicotiana tabacum* (b). Transient expression of Ds131855 was repeated according to Hunziker et al. (2021). INF1, *Phytophthora infestans* elicitin positive cell death control; EV, empty vector negative no‐cell death control. Expression of (c, e and g) hypersensitive‐response‐specific marker genes (*NbHIN1* and *NbHSR203J*) and (d, f and h) pathogen‐associated molecular pattern‐triggered immunity marker genes (*NbWRKY7* and *WRKY8*) in *N. benthamiana*. *Ds131885*, *Cm2721*, *Pp10632* and pICH86988 EV were transiently expressed in *N. benthamiana* and leaves sampled after 48 h. Means and standard errors of normalised expression values were calculated from at least three biological replicates. *p < 0.05, **p < 0.01. ***p < 0.001.

From the second set of cross‐kingdom CE proteins, Ds131885 was previously shown to elicit plant responses in *N. benthamiana* and *N. tabacum* using an *Agrobacterium tumefaciens* ‐mediated transient expression assay (ATTA) (Hunziker et al. 2021). Like Ds131885, the orthologues of this protein from *C. minus* and *P. pluvialis* also triggered defence responses in *N. benthamiana* and *N. tabacum* (Figure 3a,b). Notably, versions of Cm2721 and Pp10632 without the PR1α signal peptide for secretion to the apoplast did not trigger cell death upon transient expression in *N. tabacum* (Figure S1b,c). This suggests that secretion of these proteins to the apoplast is essential for the cell death‐inducing activity of Cm2721 and Pp10632, as previously observed for Ds131885 (Hunziker et al. 2021).

To provide further evidence that the Ds131885‐, Cm2721‐ and Pp10632‐activated chlorosis was associated with a plant defence response, the expression levels of *N. benthamiana* defence‐related marker genes were analysed by RT‐qPCR. Based on this analysis, both HR marker genes were transcriptionally upregulated at 48 hai (Figure 3c,e,g), consistent with the chlorosis and cell death triggered by these proteins in *Nicotiana* species. Both the SA and JA signalling pathways were also transcriptionally upregulated (Figure S5). The two PTI marker genes were also transcriptionally upregulated in *N. benthamiana* leaves expressing Ds131885, Cm2721 or Pp10632 (Figure 3d,f,h). The results suggest that these proteins are PAMPs that trigger plant immune responses by activating the SA‐ and JA‐mediated signalling pathways.

**FIGURE 4:**
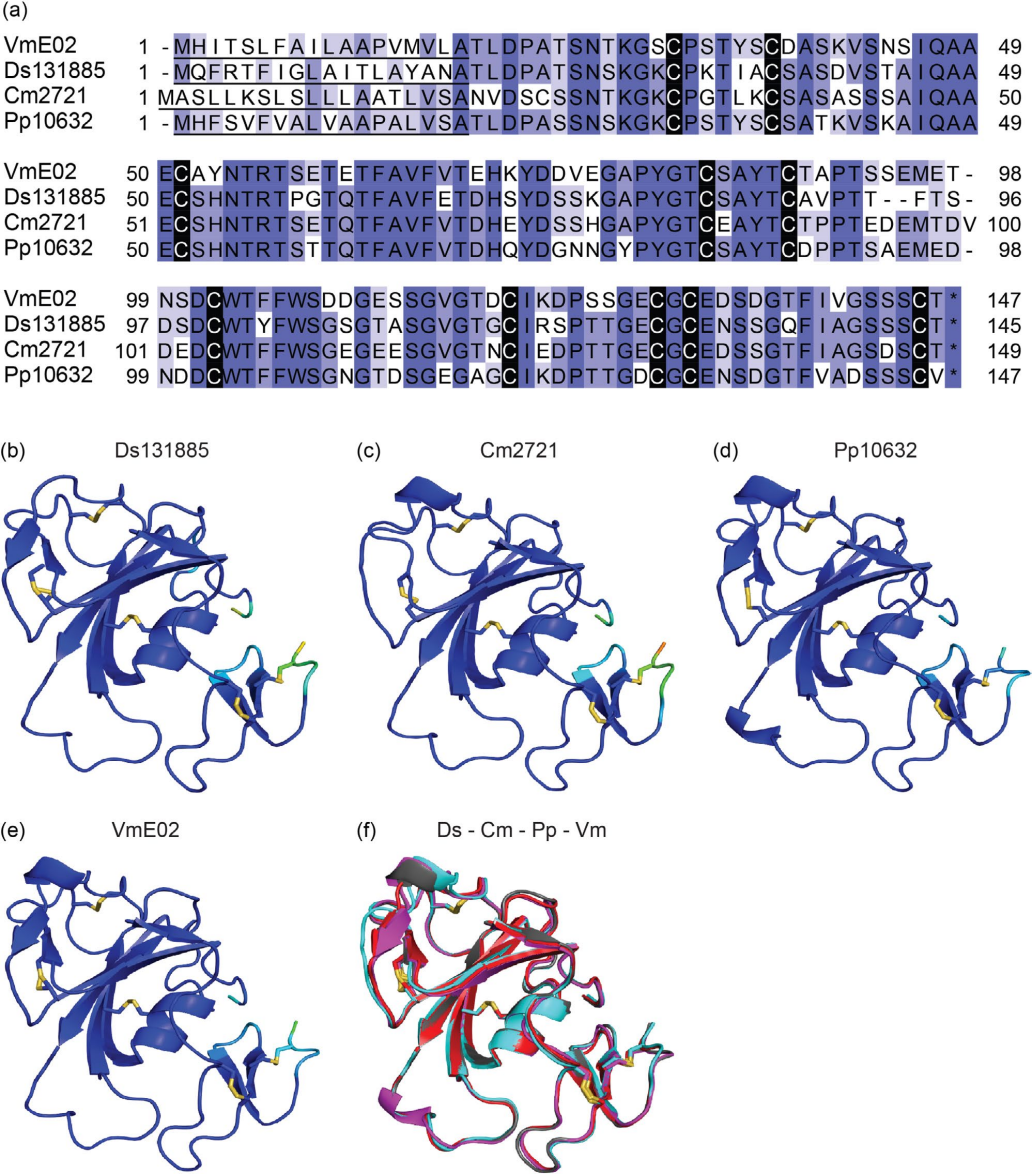
Predicted tertiary structures of Ds131885, Cm2721 and Pp10632. (a) Amino acid alignment of Ds131885, Cm2721, Pp10632 and VmE02. Cysteine residues are highlighted in black. The asterisk * indicates a stop codon. Predicted tertiary structures of Ds131885 (b), Cm2721 (c), Pp10632 (d) and VmE02 (e). For Ds131885, AlphaFold2 predicted a tertiary structure with a pLDDT score of 94.3 and a predicted TM‐score of 0.88, 94.3 and 0.88 for Cm2721, 94.7 and 0.88 for Pp10632 and 95.4 and 0.88 for VmE02. Disulphide bonds are shown as yellow sticks. (f) Alignment of predicted Ds131885 (red), Cm2721 (cyan blue), Pp10632 (magenta) and VmE02 (grey).

Based on the amino acid sequences of Ds131885, Cm2721 and Pp10632, these proteins are orthologues of VmE02 (Nie et al. 2019), a PAMP from the apple‐pathogenic fungus *V. mali* (Figure 4a). Their tertiary structures were predicted using AlphaFold2, and all four structures possess five conserved disulphide bonds (Figure 4). The top structural hit for all three proteins in the Research Collaboratory for Structural Bioinformatics Protein Data Bank (RCSB PDB) was domain II (DII) of apical membrane antigen 4 (AMA4) from *Toxoplasma gondii* (Parker et al. 2016).

**FIGURE 5:**
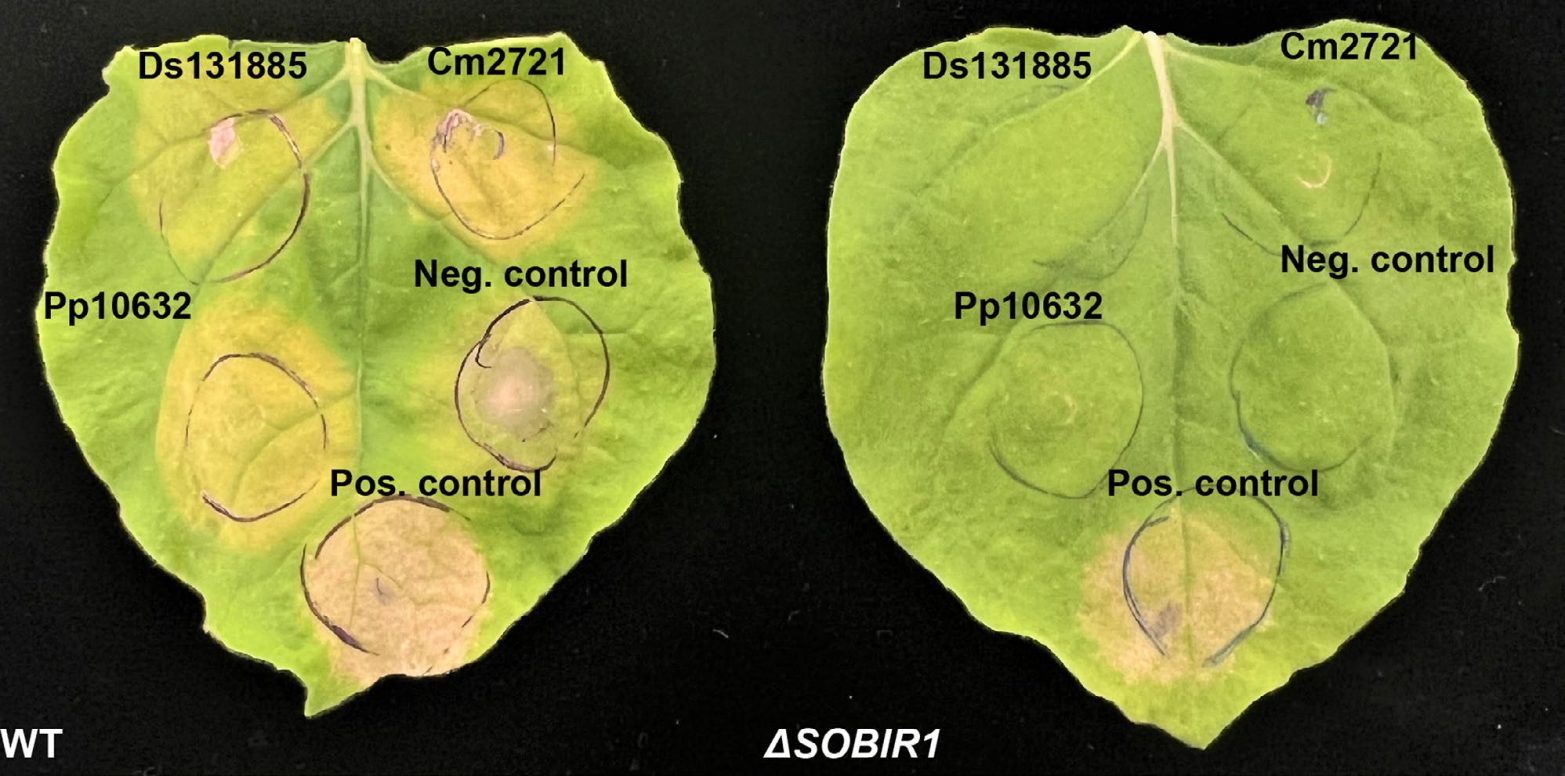
Chlorosis and cell death triggered by candidate effector proteins of *Dothistroma septosporum*, *Cyclaneusma minus* and *Phytophthora pluvialis* in *Nicotiana benthamiana* is dependent on SOBIR1. The homologous proteins Ds131885 (*D. septosporum*), Cm2721 ( *C. minus* ) and Pp10632 ( *P. pluvialis* ) were transiently expressed in wild‐type (WT) *N. benthamiana* and *N. benthamiana SOBIR1* deletion mutant (Δ*SOBIR1*). Positive (pos.) cell death control not requiring SOBIR1: *Stemphylium lycopersici* candidate effector protein TW65_01570; negative (neg.) control requiring SOBIR1 for cell death: The *Fulvia fulva* Avr9B avirulence effector protein + *Solanum lycopersicum* (tomato) Cf‐9B resistance protein (receptor‐like protein) pair.

To determine whether Ds131885, Cm2721 and Pp10632 are likely perceived by an extracellular receptor‐like protein (RLP) of the PRR class following their secretion into the apoplast, each was produced in an *N. benthamiana* line lacking the extracellular RLP co‐receptor SOBIR1 (Δ*SOBIR1*) (Huang et al. 2021) using an ATTA. As expected, the positive control protein, TW65_01570, which is a CE protein from the fungus *Stemphylium lycopersici* that is known to elicit chlorosis or cell death even in the absence of SOBIR1 (de la Rosa et al. 2024), triggered consistent chlorosis or cell death responses in both WT and Δ*SOBIR1*‐deleted *N. benthamiana*. Also, as expected, the Avr9B avirulence effector + Cf‐9B resistance protein (RLP) pair (negative control) only triggered a response in the WT (Figure 5). Curiously, Ds131885‐, Cm2721‐ and Pp10632‐triggered chlorosis was compromised in Δ*SOBIR1*‐deleted *N. benthamiana* (Figure 5), suggesting that the chlorosis triggered by these cross‐kingdom CE proteins involves recognition by one or more RLPs that require SOBIR1 as their co‐receptor.

**FIGURE 6:**
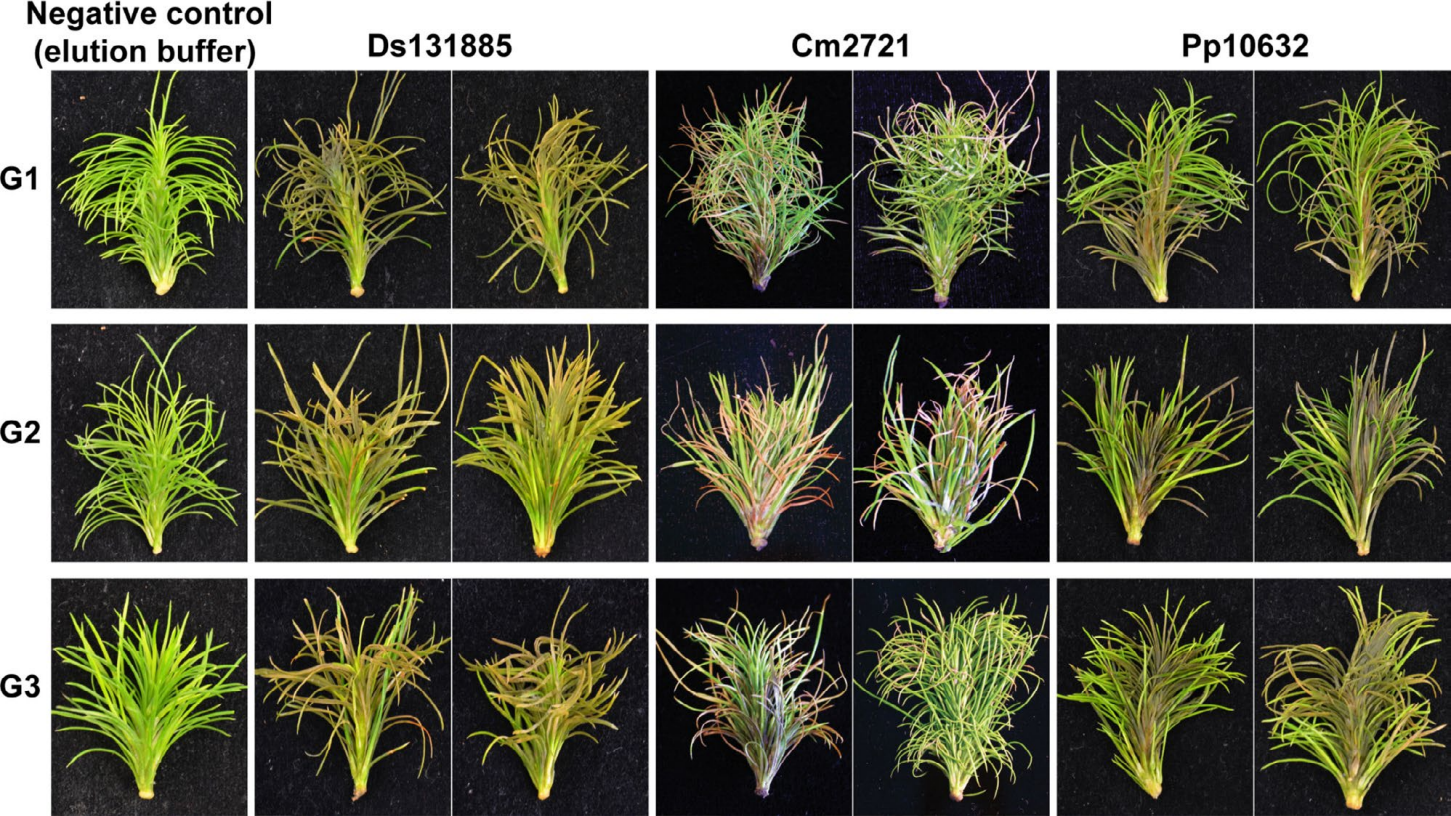
Homologous candidate effector proteins from *Dothistroma septosporum*, *Cyclaneusma minus* and *Phytophthora pluvialis* trigger cell death in clonal *Pinus radiata* shoot tissues. Whole shoots of *P. radiata* derived from family seedlots that were relatively susceptible (G1 and G2) or tolerant (G3) to *D. septosporum* infection were infiltrated with purified Ds131885, Cm2721 or Pp10632 protein produced by heterologous expression in *Pichia pastoris* at a concentration of 20 μg/mL. Elution buffer used in the protein purification step was used as a negative no‐protein control for infiltration. Representative photographs (from 12 to 24 pine shoots for each treatment) were taken 7 days after infiltration.

Because all three proteins (Ds131885, Cm2721 and Pp10632) triggered chlorosis and cell death in non‐host plants, we tested them in pine to determine whether they could induce similar responses. In all cases, as expected, the infiltration of elution buffer (negative control) did not trigger any visible damage to the shoots, while infiltration of purified proteins of Ds131885, Cm2721 and Pp10632 triggered some degree of cell death in all three pine genotypes tested (Figure 6).

We disrupted *Ds131885* to determine whether the CE it encodes is a virulence factor of *D. septosporum* during the infection of pine. Gene disruption mutants were confirmed by PCR and Southern blot hybridization (Figure S6), while the complementation strain carrying a single WT copy of *Ds131885* was confirmed by qPCR (Table S3). While the disruption of *Ds131885* did not affect the growth or sporulation of *D. septosporum* in culture, when compared to the WT fungus (Table S4), obtaining large numbers of spores required for virulence assays on *P. radiata* proved challenging (File S1). The disruption of *Ds131885* did not convincingly alter levels of *D. septosporum* fungal biomass in pine needles when compared to WT, with high variability in results for two independent disruption mutants and the complementation strain (Table 1). Details of these results are shown in File S1.

### 2.4 A Conserved β‐Trefoil Fold Is Adopted by Sequence‐Unrelated Candidate Effector Proteins From *D. septosporum*, *C. minus* and *P. pluvialis*

We previously identified two CE proteins, Ds75860 (DsEcp32‐1) from *D. septosporum* and Cm835 from *C. minus* , which trigger plant cell death responses and belong to the Ecp32 family (Tarallo et al. 2022, 2024). No homologues of this family were found in *P. pluvialis* through primary sequence searches. However, tertiary structure predictions of Ecp32 family proteins from both *D. septosporum* and *C. minus* indicated that they adopt a β‐trefoil fold. Proteins of many different phytopathogenic fungi are known to adopt this fold and, in some cases, these proteins are known to be virulence factors (Renko et al. 2012; Sabotič et al. 2019; Varrot et al. 2013).

**FIGURE 7:**
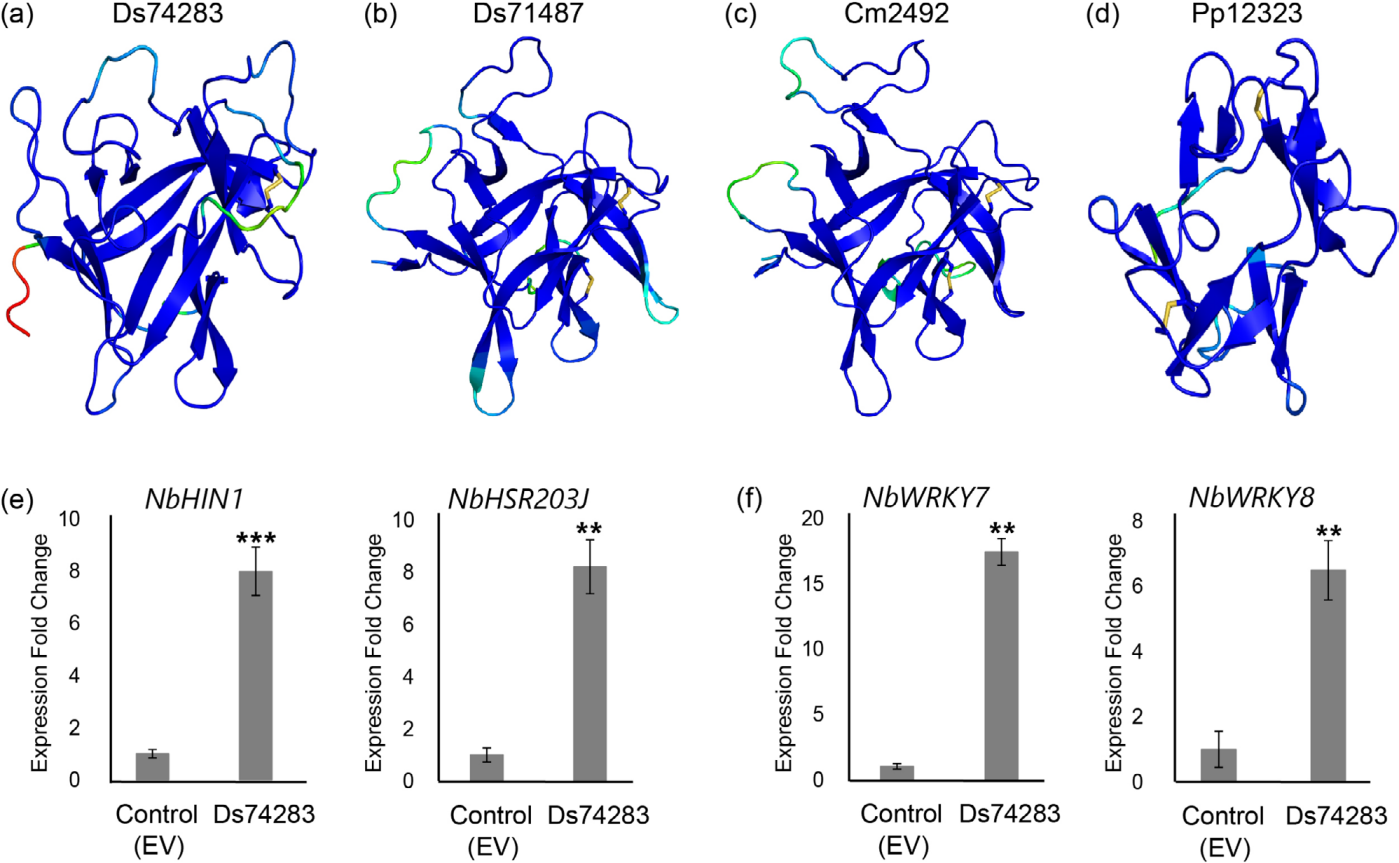
Candidate effector proteins from *Dothistroma septosporum*, *Cyclaneusma minus* and *Phytophthora pluvialis* adopt a common β‐trefoil fold. Predicted structures of (a) Ds74283 (*D. septosporum*), (b) Ds71487 (*D. septosporum*), (c) Cm2492 ( *C. minus* ) and (d) Pp12323 ( *P. pluvialis* ). For Ds74283, AlphaFold2 predicted a tertiary structure with a pLDDT score of 89.5 and a predicted TM‐score of 0.87, 91.3 and 0.88 for Ds71487, 90.8 and 0.87 for Cm2492 and 84.5 and 0.84 for Pp12323. Disulphide bonds are shown as yellow sticks. Expression of (e) hypersensitive response (HR)‐specific marker genes (*NbHIN1* and *NbHSR203J*) and (f) pathogen‐associated molecular pattern‐triggered immunity marker genes (*NbWRKY7* and *NbWRKY8*) in *Nicotiana benthamiana*. *Ds74283* and pICH86988 empty vector (EV) were transiently expressed in *N. benthamiana* and leaves sampled after 24 h. Means and standard errors of normalised expression values were calculated from at least three biological replicates. **p < 0.01, ***p < 0.001.

Three other *D. septosporum* CE proteins (Joint Genome Institute IDs Ds74283, Ds71487 and Ds69113) were also predicted to adopt a β‐trefoil fold (Figure 7a,b, Figure S7c and Table S1) and were previously tested for their cell death eliciting activity in non‐host plants, with the first two triggering cell death responses in *Nicotiana* species (Hunziker et al. 2021). Moreover, Ds74283 was also shown to trigger cell death responses in *P. radiata* (McCarthy et al. 2022). While no homologues of Ds69113 or Ds74283 were found in *C. minus* or *P. pluvialis* , a homologue of Ds71487 was found in *C. minus* (Cm2492), but not in *P. pluvialis* . Cm2492 was shown to trigger cell death in both non‐host *Nicotiana* species and was also predicted to adopt a β‐trefoil fold (Figure S7a).

The amino acid sequence similarities between all of the above‐mentioned CE proteins from *D. septosporum* and *C. minus* with a predicted β‐trefoil fold are low (ranging from 11% to 17% full‐length pairwise amino acid identity), and only structural similarity is retained between them. Alignment of the predicted tertiary structures of Ds69113, Ds71487, Ds74283 and DsEcp32‐1 indicated a conserved disulphide bond that could not be identified by sequence alignment (Figure S7d).

We next determined whether the β‐trefoil fold was present in any predicted *P. pluvialis* proteins. The AlphaFold Protein Structure database was used to identify proteins of *Phytophthora* species that are structurally similar to the β‐trefoil proteins mentioned above. Using the primary sequence of the identified *Phytophthora* proteins (Table S5), we then identified 37 *P. pluvialis* proteins through sequence similarity. Of these, 13 were classified as effectors by EffectorP, but only three were predicted to adopt a β‐trefoil fold. Only one of the three β‐trefoil proteins had a putative signal peptide: Pp12323 (Figure 7d), identified by its similarity to a *Phytophthora parasitica* structural match to Ds69113 (Table S5). Pp12323 was tested for cell death‐eliciting activity in non‐host *N. benthamiana* plants but, as found for Ds69113, Pp12323 did not elicit any visible responses (Figure S7a).

The expression of *N. benthamiana* defence genes in response to the β‐trefoil cell death‐eliciting CE protein, Ds74283, as well as Pp12323, was studied. For Ds74283, significant upregulation of all *N. benthamiana* defence genes tested occurred at 24 hai (Figures 7e,f and S8). Consistent with the ability of Ds74283 to elicit cell death, both HR‐specific marker genes, *NbHIN1* and *NbHSR203J*, were highly activated at 24 hai, along with the PTI marker genes (Figure 7e,f). Marker genes for both the SA and JA signalling pathways were also significantly upregulated in response to Ds74283 in *N. benthamiana* (Figure S8). As for Pp12323, none of the *N. benthamiana* defence genes analysed were upregulated (Figure S9). Interestingly, Ds74283‐ and DsEcp32‐1‐triggered cell death was not compromised in the Δ*SOBIR1 N. benthamiana* mutant (Figure S7b).

We previously disrupted the *Ds74283* gene in *D. septosporum* (McCarthy et al. 2022). Using these disruption mutants, we assessed whether Ds74283 contributes to virulence during infection of *P. radiata* . The results suggested reduced virulence of *Ds74283*‐disrupted strains when compared with the WT fungus (Table 1). However, no difference in fungal biomass was observed when disruption mutants were compared to the complementation strain, just as observed for the other two CE genes (Table 1, File S1). The selected transformants also had similar growth and sporulation rates in culture when compared to the WT fungus (Table S4).

## 3 Discussion

We identified core CE proteins in three foliar pine pathogens belonging to the distinct kingdoms of Fungi and Chromista; two of these sets of CE proteins are similar at both the primary sequence and tertiary structure levels, whilst one set of CE proteins is similar only at the tertiary structure level. A summary of key findings with these CE proteins is shown in Table 2.

**TABLE 2:** Summary of candidate effector proteins identified from foliar pine pathogens *Dothistroma septosporum* that have homologues in *Cyclaneusma minus* and *Phytophthora pluvialis*.

DsCE gene^a^	Ds expression (RPMK)^b^				ATTA result^c^	Predicted structure^d^	Ds CE gene disruption^e^	Predicted function^f^
	FM	Early	Mid	Late				
*69335*	425	1448	1012	553	No cell death	CAP	Lower biomass	Biotrophic effector/antimicrobial activity
*74283*	40	26	141	243	Cell death	β‐Trefoil	Lower biomass	Necrotrophic effector
*131885*	1.4	9	369	202	Cell death	VmE02‐like	No effect evident	Possible necrotrophic effector acting through RLP/SOBIR1

^a^ Joint Genome Institute (JGI) protein ID corresponding to the *Dothistroma septosporum* NZE10 (Ds) candidate effector (CE) gene of interest that was disrupted.

^b^ *D. septosporum* (Ds) gene expression during infection stages of *Pinus radiata* (fungal mycelium [FM]) (in culture), Early, Mid and Late (in planta); (Bradshaw et al. 2016) as Reads Per Million per Kilobase (RPMK).

^c^ *Agrobacterium tumefaciens* ‐mediated transient transformation assay results upon production of each CE protein in *Nicotiana* spp.

^d^ CAP, cysteine‐rich secretory proteins, antigen 5 and pathogenesis‐related 1 proteins.

^e^ Potential effect of CRISPR/Cas9 Ds CE gene disruption in *D. septosporum*. However, definite conclusions cannot be made as the complementation strains were not restored in virulence. More details are in File S1.

^f^ RLP, receptor‐like protein; SOBIR1, suppressor of BAK1‐interacting receptor‐like kinase 1.

### 3.1 CAP‐Like Proteins

The first set of shared cross‐kingdom CE proteins, Ds69335 (*D. septosporum*), Cm8840 ( *C. minus* ) and Pp7927 ( *P. pluvialis* ), have strong sequence and structural similarity with proteins of the CAP superfamily. The high level of structural similarity between the CAP domains of Ds69335, Cm8840 and Pp7927 suggests that these CEs may have the same biological function in planta. None of the abovementioned CAP proteins triggered cell death responses in *N. benthamiana*, although Ds69335 induced transcriptional upregulation of two PTI marker genes in this non‐host.

All three proteins are structurally similar to the CAP protein Pry1, from *S. cerevisiae* , which in turn is structurally similar to pathogenesis‐related protein 1 (PR‐1) from plants. CAP family members are known to bind lipids and sterols, including toxic hydrophobic compounds, suggesting a possible detoxification role for CAP proteins (Choudhary and Schneiter 2012; Darwiche et al. 2017). In line with CAP proteins from other pathogenic fungi and oomycetes (Darwiche et al. 2017; Jiang et al. 2023), *Ds69335* is also highly upregulated at the early infection stage of pine by *D. septosporum*, which could suggest this protein, and perhaps its homologues Cm8840 and Pp7927, might act as biotrophic effectors that suppress host defence responses by detoxification of hydrophobic compounds. UmPR‐1La, from the corn smut fungus *Ustilago maydis* , was shown to bind phenolic compounds, thereby protecting fungal hyphae against toxic plant phenolics, and also suppressing plant defence responses (Lin et al. 2023). While some CAP proteins function in the apoplast (Jiang et al. 2023; Mesarich et al. 2018), others function inside host cells. CcCAP1 from the fungal tree pathogen *Cytospora chrysosperma* localises to the plant nucleus where it is thought to modulate plant immunity (Han et al. 2021).

Whether Ds69335 is required for the full virulence of *D. septosporum* in pine is not clear. Apart from one outlier, all replicates across two independent *Ds69335* disruption mutants showed decreased biomass in planta when compared to the WT fungus, suggesting a decrease in virulence. Lower fungal biomass could be due to activation of the pine immune system in the absence of the Ds69335 protein or possibly due to host defences no longer being suppressed in the absence of Ds69335. However, there was considerable variation between biological replicates and limits on the numbers of replicates that could be tested in our system. Moreover, the complementation strain was not restored to WT levels of virulence, so we cannot be certain that the lower virulence phenotypes in the disrupted strains were due to those specific gene mutations. The WT gene introduced into the complemented strain might have been mutated following Cas9 genome integration and expression, as has been reported previously (Rocafort, Arshed, et al. 2022). More details and further discussion of the *D. septosporum* virulence assays are in File S1.

### 3.2 VmE02‐Like Proteins

The second set of shared cross‐kingdom CE proteins, Ds131885 (*D. septosporum*), Cm2721 ( *C. minus* ) and Pp10632 ( *P. pluvialis* ), are orthologues of VmE02, a cross‐kingdom PAMP first identified in the apple‐pathogenic fungus, *V. mali*, which triggers cell death in multiple plant species, including *N. benthamiana*, tomato, pepper and apple, and is highly conserved in different *Phytophthora* and fungal species, including saprophytes and endophytes (Nie et al. 2019). In this study, the three proteins from the pine pathogens also triggered cell death in non‐host and host plants. Moreover, these proteins activated immune responses in *N. benthamiana*, including the expression of PTI‐marker genes, suggesting their recognition as a PAMP. The cell death triggered by Ds131885, Cm2721 and Pp10632 was compromised in *N. benthamiana* plants in which the gene coding for SOBIR1, an extracellular immune system co‐receptor for RLPs (Huang et al. 2021), was deleted. This suggests that these proteins might be recognised by extracellular RLP immune receptors.

VmE02‐triggered cell death requires the *N. benthamiana* RLP immune receptor RE02 (Nie et al. 2019, 2020). Due to their strong primary sequence and predicted structural similarity, it is likely that the orthologous proteins from the three pine pathogens are also recognised by this same receptor. RE02 is the best BLASTp hit of PsRLK, a characterised immune receptor from *Pinus sylvestris* (Ávila et al. 2006), suggesting the *Pinus* genus might also encode an immune receptor that could potentially recognise Ds131885, Cm2721 and Pp10632. The presence of an RE02 homologue in *P. radiata* could explain the cell death observed in both non‐host (*N. benthamiana*) and host ( *P. radiata* ) species following protein delivery. The gene encoding this receptor could potentially be identified in *P. radiata* as a possible *R* gene for use in selecting or engineering disease resistance, provided the pathogens do not use this receptor to switch to or enhance necrotrophy. Alternatively, if recognition by this receptor facilitates pathogen infection at the necrotrophic stage, it will be considered a susceptibility (*S*) gene. Mutation or deletion of *S* genes from the host can limit the ability of the pathogen to cause disease, something that would also be beneficial in breeding programmes (van Schie and Takken 2014). This could be determined by deletion or silencing of either the host receptor gene or the pathogen effector gene. Deletion of *VmE02* from *V. mali*, the orthologue of *Ds131885*, generated mutants that had the same filamentous growth as the WT fungus, suggesting that *VmE02* does not affect the virulence of *V. mali* in apple leaves (Nie et al. 2019). Similarly, our virulence assays with *Ds131885* mutants in pine showed no evidence for a role in virulence but were inconclusive. In future, silencing or disrupting the putative host receptor gene for Ds131885 could reveal if it is involved in resistance or susceptibility to disease.

### 3.3 β‐Trefoil‐Like Proteins

Proteins with a β‐trefoil fold have low or no sequence similarity, with conservation only observed for some hydrophobic residues (Feng et al. 2010; Kirioka et al. 2017). In line with this lack of sequence similarity, a β‐trefoil fold was found to be associated with sequence‐unrelated CE proteins from the three pine pathogens. Of note, however, the β‐trefoil fold adopted by the CE of *P. pluvialis* (Pp12323) was quite distinct from the β‐trefoil fold adopted by the CE proteins of *D. septosporum* and *C. minus* , suggesting that the observed structural similarity across the two kingdoms is weak. The strong structural similarity was, however, observed between the β‐trefoil proteins identified from the two fungi, with all predicted to possess a conserved disulphide bond that is absent from Pp12323 of *P. pluvialis* . This conserved disulphide bond is anticipated to be important for the structural maintenance and/or stability of the overall fold in *D. septosporum* and *C. minus* . Recently, the β‐trefoil fold was found to be widespread in fungal species (Derbyshire and Raffaele 2023). If indeed the β‐trefoil fold is common amongst effectors of fungal and oomycete species, it would be interesting to determine the biological function and/or host targets of these proteins, and if this function/target is also conserved, as it may provide further support for an ancestral origin.

Some proteins with conserved structural folds display different rather than shared functions, and their structural conservation simply provides a template for the diversification of functions. β‐Trefoil proteins are associated with plant–pathogen interactions, having different functions during host infection (Žurga et al. 2015). The β‐trefoil fold is present in protease inhibitors (Renko et al. 2012). Fungal pathogens often target host proteases involved in plant defence by secreting effectors that serve as protease inhibitors (Mueller et al. 2013; van Esse et al. 2008), which suggests these β‐trefoil proteins might be involved in suppressing plant defence responses. However, other proteins with a β‐trefoil fold, like cytolysins and some lectins, are known to interact with and disrupt the host plasma membrane, leading to host cell death (Juillot et al. 2016; Schubert et al. 2012; Wohlschlager et al. 2011). This could facilitate a pathogen's necrotrophic phase and explain cell death responses such as those observed in this work with non‐host and host plants (in both susceptible and tolerant pine genotypes).

Interestingly, all CE proteins from *D. septosporum* that are predicted to have a β‐trefoil fold are encoded by genes that are upregulated during the late (necrotrophic) stage of pine infection (when compared with in vitro, early and mid stages) (Bradshaw et al. 2016). Ds74283 might therefore be a necrotrophic effector, facilitating disease lesion expansion by destruction of plant cells. Indeed, two independent *Ds74283* mutants showed a clear decrease in fungal biomass in planta, but problems with the complementation strain, as discussed for the *Ds69335* mutant above, meant that no definitive conclusion could be drawn from these results.

Overall, these results support the premise that many sequence‐unrelated effector proteins share conserved folds, likely evolving from common ancestral proteins (de Guillen et al. 2015). Sequence diversification can lead to amino acid polymorphisms while maintaining the overall structure. Because of the constant arms race between pathogens and plants, it is not surprising that pathogens would have large structurally similar families of virulence factors; a recognised effector of such a family could then be lost without an associated fitness cost to the pathogen, while other sequence‐unrelated family members can acquire new virulence functions or escape host recognition.

### 3.4 Conclusion

Although results from the virulence assays were inconclusive, two proteins described here could potentially be important when *D. septosporum* infects the host *P. radiata*, based on their known expression levels in planta, similarity to well characterised virulence factors, expression of PTI markers in a non‐host plant and, in the case of Ds74283 and Ds131885, their ability to cause cell death in pine (Table 2). This could be evaluated through RNAi‐based silencing of these virulence factors, which is an effective control strategy against some pathogens (Song and Thomma 2018; Wang et al. 2016). Moreover, the proteins identified in this study are shared between pine pathogens *D. septosporum*, *C. minus* and *P. pluvialis* (Table 2), although we cannot affirm that the β‐trefoil fold from *P. pluvialis* is shared between the two other fungi. Despite that, there seems to be evidence suggesting that these highly diverse pathogens might use similar tactics to infect and subdue their host. Identifying common structural folds shared between proteins from these pine pathogens can help to better understand the mechanisms they employ to successfully infect the host. Host defences based on recognition of these highly conserved effectors are more likely to be durable as there would likely be a fitness penalty associated with their mutation or loss. Thus, analysis of existing and new potential shared CEs between these three pine pathogens can lead to the identification of *P. radiata* genes that could be used for the breeding or engineering of disease resistance. This could not only target pathogens currently impacting plantations, but also other potential threats that have these homologous effectors, for example, *Lecanosticta acicola* (Hunziker et al. 2021), which is not currently present in New Zealand, but is impacting *P. radiata* forests in many other parts of the world (van der Nest et al. 2019). The association of a putative immune receptor from pine with resistance to oomycete and fungal pathogens will need to be evaluated in the forest and then molecular markers based on those receptors could be developed for breeding durable and broad‐spectrum resistance.

## 4 Experimental Procedures

### 4.1 Microorganisms and Plants

*Dothistroma septosporum* NZE10 (de Wit et al. 2012), *C. minus* NZFS809 (Tarallo et al. 2024) and *P. pluvialis* NZFS3000 (Studholme et al. 2016) were used in this study. *Escherichia coli* DH5α (Taylor et al. 1993), *A. tumefaciens* GV3101 (Holsters et al. 1980) and *Pichia pastoris* GS115 (Invitrogen) were used for gene cloning, ATTAs and heterologous protein production, respectively. *N. tabacum* ‘Wisconsin 38’, as well as WT and Δ*SOBIR1 N. benthamiana* (Huang et al. 2021) were used as model non‐host plants for ATTAs. Clonal shoots of *P. radiata* derived from family seedlots that were either relatively susceptible (G1 and G2) or relatively tolerant (G3) to *D. septosporum* infection were used for protein infiltrations, while WT *P. radiata* seedlings derived from a cross between two parents with similarly low levels of tolerance to *D. septosporum* were used in virulence assays.

### 4.2 Characterisation of Shared Candidate Effector Proteins From *D. septosporum*, *C. minus* and *P. pluvialis*

CE proteins of *D. septosporum* investigated in this study were identified previously (Bradshaw et al. 2016; Hunziker et al. 2021). Reciprocal tBLASTn (translated genome) and BLASTp (protein catalogue) searches were used to identify homologues of *D. septosporum* CE proteins (E‐value < 10^−5^) in *C. minus* NZFS809 (Tarallo et al. 2024) and *P. pluvialis* NZFS3000 (Studholme et al. 2016). Protein sequence alignments, phylogenetic trees, protein tertiary structure predictions and identification of structural homologues were performed according to Tarallo et al. (2022). The Foldseek server (https://search.foldseek.com/search; van Kempen et al. 2024) was used to identify similarities in tertiary structure between AlphaFold2‐predicted structures of *D. septosporum* CE proteins and proteins of all *Phytophthora* species present in the AlphaFold Protein Structure database (Varadi et al. 2022). The amino acid sequence of the top hit obtained by querying each predicted *D. septosporum* CE protein structure was used to search the predicted proteome of *P. pluvialis* . *P. pluvialis* proteins predicted to have a β‐trefoil fold were classified using EffectorP v. 3.0 (Sperschneider and Dodds 2021). IDRs were predicted in CE proteins using the Predictor of Natural Disordered Regions (PONDR) server (prediction score ≥ 0.8) (http://www.pondr.com/; Romero et al. 2001).

### 4.3 Candidate Effector Expression for Functional Characterisation in Non‐Host and Host Plants

#### 4.3.1 *Agrobacterium tumefaciens* ‐Mediated Transient Transformation Assays

Nucleotide sequences encoding full‐length mature CE proteins were either amplified from genomic DNA (gDNA) using primers shown in Table S6 or synthesised directly into the expression vector by Twist Bioscience (San Francisco, CA, USA). The PCR products, along with an entry vector holding a fused PR1α signal peptide (SP) (for secretion to the apoplast) and a 3 × FLAG‐tag (detection by western blotting) or N‐3 × FLAG‐tag (versions without SP), were used as entry modules for Golden Gate assembly (Engler et al. 2009) into the binary expression vector pICH86988 (Weber et al. 2011). Verified CE expression vectors were transformed into *A. tumefaciens* GV3101 as described by Guo et al. (2020). Overnight cultures of transformed *A. tumefaciens* were resuspended in 1 mL of buffer (10 mM MgCl_2_, 10 mM MES‐KOH, 100 μM acetosyringone [Sigma‐Aldrich]) and infiltrated into *Nicotiana* leaves at a final OD_600_ of 1.0 (Ma et al. 2012). The elicitin INF1 from *Phytophthora infestans* (Kamoun et al. 1997) was used as a positive cell death control, while empty pICH86988 was used as a negative cell death control. *S. lycopersici* TW65_01570 was used as a positive cell death control in the *N. benthamiana* Δ*SOBIR1* deletion line, while the Avr9B/Cf‐9B pair (de la Rosa et al. 2024) was used as a negative cell death control.

#### 4.3.2 RNA Extraction and Quantitative Reverse Transcription PCR Analysis

*Nicotiana benthamiana* leaves were infiltrated with the *Agrobacterium* strain transformed with each *D. septosporum*, *C. minus* or *P. pluvialis* CE gene as above, along with the empty pICH86988 vector. Samples were collected 24 and 48 hai, and then *N. benthamiana* total RNA was extracted with an RNeasy Plant Mini Kit (Qiagen). The concentration and purity of RNA were estimated with a NanoDrop (Nanodrop Technologies Inc.). For cDNA synthesis, 1 μg of total RNA from each sample was used, and synthesis was performed with a QuantiTect Reverse Transcription Kit (Qiagen), according to the manufacturer's instructions. Quantitative PCR (qPCR) was performed with SensiFAST SYBR No‐ROX mix (Meridian Bioscience) in a LightCycler 480 III (Roche). Relative expression of *N. benthamiana* defence genes was calculated with the Q‐Gene method (Muller et al. 2002) with three independent biological replicates. Transcript levels were normalised to the reference gene *NbActin* (Sainsbury and Lomonossoff 2008) and compared to the empty vector control (set as 1). Means and standard errors were calculated from at least three biological replicates. Results were statistically analysed using Student's *t* test. All primers are shown in Table S6.

#### 4.3.3 *Pinus radiata* Infiltration With Purified Candidate Effector Proteins

The same CE nucleotide sequences used to generate the ATTA expression vectors mentioned above were ligated into the expression vector pPic9‐His_6_ (Invitrogen) and used to transform *P. pastoris* GS115, according to Kombrink (2012). Heterologous expression of CE proteins in *P. pastoris* was performed as previously described by Weidner et al. (2010). Secreted CE proteins were purified from the culture filtrate using a Ni Sepharose 6 Fast Flow resin (GE Healthcare) based on immobilised metal ion affinity. Proteins were then vacuum‐infiltrated into tissue‐cultured *P. radiata* shoots according to Hunziker et al. (2021).

### 4.4 CRISPR/Cas9 Gene Disruption of *D. septosporum* NZE10 Candidate Effectors and Functional Characterisation

#### 4.4.1 Vector Construction, Targeted Gene Disruption and Complementation

To disrupt the *D. septosporum* CE genes *Ds69335*, *Ds74283* and *Ds131885*, two different plasmids were generated as described by McCarthy et al. (2022): one containing a single‐guide RNA (sgRNA) sequence complementary to the target sequence and one with donor DNA (dDNA) as a template for gene disruption by homologous recombination (details of flanking sequences for each gene are in Table S7). Complementation plasmids were created by amplifying the complete *Ds69335*, *Ds74283* and *Ds131885* coding regions, along with 700 bp of upstream (5′ flank) for the three genes and at least 370 bp of downstream (3′ flank) gDNA sequence (details in Tables S6 and S7). Each amplified PCR product was digested with appropriate enzymes and ligated into the pBC‐phleo vector, which confers phleomycin resistance to the complementation transformants (Chettri et al. 2012).

Disruption and complementation strains were obtained by protoplast‐mediated transformation (Bradshaw et al. 1997) with the respective plasmids transformed into *D. septosporum* WT and transformant strains, respectively, then confirmed by PCR and Southern blot hybridization, as previously described (McCarthy et al. 2022). qPCR was performed to determine the copy number of complementation strains. A known *D. septosporum* single‐copy gene, *DsAflR* (Chettri et al. 2013), was used as a reference, and the ratio between the target gene and the *DsAflR* gene was calculated using cycle threshold (*C*_t_) values.

#### 4.4.2 Growth and Sporulation Phenotype Characterisation

Mycelia of actively growing colonies of *D. septosporum* transformants were cut with a 5 mm diameter sterile cork borer, and three plugs were placed on each of three replicate Dothistroma medium (DM) plates (Bradshaw et al. 2000) for each transformant and WT fungus. After incubation at 22°C for 3 weeks, radial colony growth was measured along two perpendicular axes and calculated as mm/day. Sporulation was assessed by spreading 50 μL spore suspension (10^5^ spores/mL) on pine extract minimal medium with glucose (PMMG) plates (McDougal et al. 2011) with three replicate plates for each strain. After incubation at 22°C for 7 days, 2 mL of sterile distilled water was added to each plate and incubated for 10 min at room temperature. Spores were then suspended using a sterile glass spreader, and the spore concentration was quantified using a cytometer (Weber Scientific).

#### 4.4.3 Virulence Assays and Fungal Biomass Analysis

For inoculation on *P. radiata* , spores from two independent disruption strains and one complementation strain, as well as the WT fungus, were obtained as above and sprayed on approximately 16‐month‐old *P. radiata* seedlings. Two sets of experiments were performed at two separate times, and each had its own WT control, with four replicate plants used for each transformant. Inoculated seedlings were maintained in misting chambers as described by Kabir et al. (2013). After 10 weeks, needles were sampled and the relative fungal biomass present in pine needle lesions was estimated using qPCR to determine the relative levels of the single‐copy *D. septosporum* polyketide synthase A (*DsPksA*) gene and the *P. radiata* reference gene cinnamyl alcohol dehydrogenase (*CAD*) in each sample, as previously described (Chettri et al. 2012). The fungal biomass was normalised by the dry weight (DW) of each sample. Further details on the experimental set‐up can be found in File S1.

## Conflicts of Interest

The authors declare no conflicts of interest.

## Supporting information


**Figure S1.** (a) Western blot detection of Ds69335 protein from *Dothistroma septosporum* that did not trigger chlorosis or cell death when expressed in *Nicotiana benthamiana*. (b) Proteins that previously triggered consistent cell death in non‐host *Nicotiana tabacum* (Nt), Cm2721 and Pp10632, were expressed without a signal peptide (ΔSP) using an *Agrobacterium tumefaciens* ‐mediated transient expression assay to assess their ability to elicit cell death. INF1, *Phytophthora infestans* elicitin positive cell death control; EV, empty vector negative no‐cell death control. Photographs were taken 7 days after infiltration. (c) Western blot detection of Cm2721 and Pp10632 that lack the PR1α signal peptide (ΔSP). Anti‐FLAG antibody was used for immunodetection. Marker on the left of each membrane was the PageRuler Prestained Protein Ladder (ThermoScientific). Asterisks (*) refer to the protein bands.

**Figure S2.** Expression of *Nicotiana benthamiana* defence‐related genes in response to production of Ds69335, Cm8840 and Pp7927. Expression of defence‐related marker genes (a, d and g) *NbPR1a* and *NbPR2*, salicylic acid‐dependent immunity; (b, e and h) *NbPR4* and *NbLOX*, jasmonic acid‐dependent immunity; (c, f and i) *ERF1*, ethylene‐dependent immunity in *N. benthamiana*. Ds69335, Cm8840, Pp7927 and empty pICH86988 vector (EV) were expressed in *N. benthamiana* using an *Agrobacterium tumefaciens* ‐mediated transient expression assay and leaves were sampled after 48 h. Transcript levels were normalised to the reference gene *NbActin* and compared to the level of the control (set as 1). Means and standard errors were calculated from at least three biological replicates.

**Figure S3.** Predicted protein tertiary structures of Ds69335 from *Dothistroma septosporum*, Cm8840 from *Cyclaneusma minus* and Pp7927 from *Phytophthora pluvialis*. Predicted structures of (a) Ds69335, (b) Cm8840 and (c) Pp7927. Protein structures were predicted with AlphaFold2, rendered in PyMol v. 2.5 (DeLano, 2002; Jumper et al., 2021; Mirdita et al., 2022) and coloured according to their AlphaFold2 pLDDT score: dark blue for regions predicted with high confidence, light blue and green for regions of low confidence and red for very low confidence regions. Disulphide bonds and/or cysteine residues are shown as yellow sticks. In Ds69335, cysteine residues at positions 150 and 230, and at positions 225 and 248, likely form disulphide bonds. The same is also likely for cysteine residues at positions 103 and 183 of Cm8840. (d) Characterised structure of Pry1 from *Saccharomyces cerevisiae* (Research Collaboratory for Structural Bioinformatics protein data bank [RCSB PBD] ID: 5jys) (Darwiche et al. 2016).

**Figure S4.** *Dothistroma septosporum* CRISPR/Cas9 *Ds69335* disruption and confirmation by PCR and Southern hybridization. (a) Schematic diagram showing the disruption of *Ds69335* and insertion of the *nptII* geneticin resistance gene cassette (P*trpC*‐*nptII*‐T*trpC*) through homologous recombination, using donor DNA (dDNA) as template. The dDNA was constructed with two flanks (5′ and 3′) from *Ds69335*, starting 3 bp from the double‐strand break (shown as the vertical black line crossing the gene), with the *nptII* cassette in the middle. Positions of primers are illustrated by grey flags with the primer name above. Also shown are the restriction enzyme sites and probe binding site (red line) used for Southern hybridization and the fragment sizes expected from the disruption of *Ds69335* by insertion of the *nptII* cassette. (b) PCR amplicons generated with primers MT91 and MT92, which bind to the start and stop regions of the coding sequence, respectively. *Ds69335* mutants should have a product of 3.6 kb, while the wild‐type (WT) should have one of 0.89 kb. (c) PCR amplicons generated with primers MT93 and MT94, which bind either side of the target genomic region. *Ds69335* mutants should have a product of 5.4 kb, and the WT fungus 2.6 kb. Relevant size labels are shown on the right of each gel. (d) Southern hybridization of *Stu*I and *Sma*I‐digested gDNA from *D. septosporum* WT fungus and six candidate *Ds69335* mutants (1, 2, 4, 7, 36, 40) with *Sma*I and five with *Stu*I (2, 4, 7, 36, 40) using a digoxigenin (DIG)‐11‐dUTP‐labelled probe binding to the 3′ flank region of *Ds69335* that was present in the dDNA. Expected fragment sizes are marked with a red asterisk for *Ds69335* mutants and black for WT fungus. Each of the five *Ds69335* mutants were sampled from independent transformation plates. (e) PCR screening of putative *Ds69335*‐T2 complementation strains with primers MT91 and MT92, which bind to the start and stop of the coding sequence, respectively. Complementation strains should have a product of 0.89 kb, the same as the WT fungus, and a 3.6 kb from the *nptII* cassette insertion. Lanes C1–C4 show the complementation strains, lane 2 shows the *Ds69335*‐T2 mutant.

**Figure S5.** Expression of *Nicotiana benthamiana* defence‐related genes in response to production of Ds131885, Cm2721 and Pp10632. Expression of defence‐related marker genes (a, d and g) *NbPR1a* and *NbPR2*, salicylic acid‐dependent immunity; (b, e and h) *NbPR4* and *NbLOX*, jasmonic acid‐dependent immunity; (c, f and i) *ERF1*, ethylene‐dependent immunity in *N. benthamiana*. Ds131885, Cm2721, Pp10632and empty pICH86988 vector (EV) were expressed in *N. benthamiana* using an *Agrobacterium tumefaciens* ‐mediated transient expression assay and leaves were sampled after 48 h. Transcript levels were normalised to the reference gene *NbActin* and compared to the level of the control (set as 1). Means and standard errors were calculated from at least three biological replicates. *p < 0.05, **p < 0.01, ***p < 0.001.

**Figure S6.** *Dothistroma septosporum* CRISPR/Cas9 *Ds131885* disruption and confirmation by PCR and Southern hybridization. (a) Schematic diagram showing the disruption of *Ds131885* and insertion of the *nptII* geneticin gene cassette (P*trpC*‐*nptII*‐T*trpC*) through homologous recombination, using donor DNA (dDNA) as template. The dDNA was constructed with two flanks (5′ and 3′) from *Ds131885*, starting 3 bp from the double‐strand break (shown as the vertical black line crossing the gene), with the *nptII* cassette in the middle. Positions of primers are illustrated by grey flags with the primer name above. Also shown are the restriction enzyme sites and probe binding site (red line) used for Southern hybridization and the fragment sizes expected from the disruption of *Ds131885* by insertion of the *nptII* cassette. (b) PCR amplicons generated with primers MT99 and MT100, which bind to the start and stop regions of the coding sequence, respectively. *Ds131885* mutants should have a product of 3.3 kb, and the wild‐type (WT) fungus 0.54 kb. (c) PCR amplicons generated with primers MT101 and MT102, which bind on either side of the target genomic region. *Ds131885* mutants should have a product of 4.7 kb, and the WT fungus 1.9 kb. Relevant size labels are shown on the right of each gel. (d) Southern hybridization of EcoRI and NdeI‐digested gDNA from *D. septosporum* WT fungus and six candidate *Ds131885* mutants, 2, 9, 13, 25, 46 and 47, using a digoxigenin (DIG)‐11‐dUTP‐labelled probe binding to the 3′ flank region of *Ds131885* that was present in the dDNA. Expected fragment sizes are marked with a red asterisk for *Ds131885* mutants and black for WT fungus. Each of the six *Ds131885* mutants were sampled from independent transformation plates. (e) PCR screening of putative *Ds131885*‐T9 complementation strains with primers MT99 and MT100, which bind to the start and stop of the coding sequence, respectively. Complementation strains should have a product of 0.54 kb, the same as the WT fungus, and 3.3 kb from the *nptII* cassette insertion. Lanes C1‐C4 show the complementation strains, lane 9 shows the *Ds131885*‐T9 mutant.

**Figure S7.** Features of β‐trefoil proteins from *Dothistroma septosporum*, *Cyclaneusma minus* and *Phytophthora pluvialis*. (a) Proteins Cm2492 from *C. minus* and Pp12323 from *P. pluvialis* were expressed in *Nicotiana benthamiana* (top) and *Nicotiana tabacum* (lower) using an *Agrobacterium tumefaciens* ‐mediated transient expression assay (ATTA) to assess their ability to elicit chlorosis or cell death. Representative images are shown (*n* = 12–24 infiltration zones), from at least three independent experiments. INF1, *Phytophthora infestans* elicitin positive cell death control; EV, empty vector negative no‐cell death control. Photographs were taken 7 days after infiltration. (b) Proteins Ds74283 and DsEcp32‐1 from *D. septosporum* were expressed in wild‐type (WT) *N. benthamiana* and a *N. benthamiana* SOBIR1 deletion mutant (Δ*SOBIR1*) using an ATTA to assess their ability to elicit chlorosis or cell death. Representative images are shown (*n* = 12–24 infiltration zones), from at least three independent experiments. Positive (pos.) cell death control not requiring SOBIR1: *Stemphylium lycopersici* TW65_01570; negative (neg.) control requiring SOBIR1 for cell death: Avr9B/Cf‐9B. Photographs were taken 7 days after infiltration. (c) Predicted structure of Ds69113; AlphaFold2 predicted a tertiary structure with a pLDDT score of 90.7 and a predicted TM‐score of 0.88. (d) Alignment of predicted structures of *D. septosporum* β‐trefoil proteins Ds69113 (blue), Ds71487 (black), Ds74283 (grey) and DsEcp32‐1 (purple). The conserved disulphide bond is shown as red sticks.

**Figure S8.** *Dothistroma septosporum* candidate effector Ds74283 triggers the expression of plant defence response genes in *Nicotiana benthamiana*. Expression of defence‐related marker genes (a) *NbPR1a* and *NbPR2*, salicylic acid (SA)‐dependent immunity; (b) *NbPR4* and *NbLOX*, jasmonic acid (JA)‐dependent immunity; (c) *ERF1*, ethylene‐dependent immunity in *N. benthamiana*. Ds74283 and empty pICH86988 vector (EV) were expressed in *N. benthamiana* using an *Agrobacterium tumefaciens* ‐mediated transient expression assay and leaves were sampled after 24 h. Transcript levels were normalised to the reference gene *NbActin* and compared to the level of the control (set as 1). Means and standard errors were calculated from at least three biological replicates. *p < 0.05, **p < 0.01.

**Figure S9.** Expression of *Nicotiana benthamiana* defence‐related genes in response to production of Pp12323. Expression of (a) hypersensitive‐response (HR)‐specific marker genes (*NbHIN1* and *NbHSR203J*); (b) pathogen associated molecular pattern immunity (PTI) marker genes (*NbWRKY7* and *WRKY8*) and defence‐related marker genes; (c) *NbPR1a* and *NbPR2*, salicylic acid (SA)‐dependent immunity; (d) *NbPR4* and *NbLOX*, jasmonic acid (JA)‐dependent immunity; (e) *ERF1*, ethylene‐dependent immunity) in *N. benthamiana*. Pp12323 and empty pICH86988 vector (EV) were expressed in *N. benthamiana* using an *Agrobacterium tumefaciens* ‐mediated transient expression assay and leaves were sampled after 48 h. Transcript levels were normalised to the reference gene *NbActin* and compared to the level of the control (set as 1). Means and standard errors were calculated from at least three biological replicates.

**File S1.** Virulence assays of *Dothistroma septosporum* transformants in *Pinus radiata* .

**Table S1.** *Dothistroma septosporum* NZE10 candidate effector proteins (DsCEs) and their homologues in *Cyclaneusma minus* NZFS809 and *Phytophthora pluvialis* NZFS3000.

**Table S2.** Sequence features of *Dothistroma septosporum*, *Cyclaneusma minus* and *Phytophthora pluvialis* proteins.

**Table S3.** Copy number determination of *Dothistroma septosporum* NZE10 complementation strains.

**Table S4.** Growth rate and sporulation assays involving candidate effector gene disruption and complementation strains of *Dothistroma septosporum*.

**Table S5.** Searches for *Phytophthora* β‐trefoil proteins using the Foldseek server.

**Table S6.** Primers used in this study.

**Table S7.** *Dothistroma septosporum* sequence details for flanks used to generate donor DNA for homologous recombination and complementation strains.

## Data Availability

The data that support the findings of this study are available in the File Supporting Information of this article.
