# Supplementary material for: Foliar Pine Pathogens From Different Kingdoms Share Defence‐Eliciting Effector Proteins
Source: Mol Plant Pathol. 2025 Mar 2;26(3):e70065. doi: 10.1111/mpp.70065 (PMC11872807; doi:10.1111/mpp.70065)
Supplement: Supplementary file 1 — Figure S1. (a) Western blot detection of Ds69335 protein from Dothistroma septosporum that did not trigger chlorosis or cell death when expressed in Nicotiana benthamiana. (b) Proteins that previously triggered consistent cell death in non‐host Nicotiana tabacum (Nt), Cm2721 and Pp10632, were expressed without a signal peptide (ΔSP) using an Agrobacterium tumefaciens ‐mediated transient expression assay to assess their ability to elicit cell death. INF1, Phytophthora infestans elicitin positive cell death control; EV, empty vector negative no‐cell death control. Photographs were taken 7 days after infiltration. (c) Western blot detection of Cm2721 and Pp10632 that lack the PR1α signal peptide (ΔSP). Anti‐FLAG antibody was used for immunodetection. Marker on the left of each membrane was the PageRuler Prestained Protein Ladder (ThermoScientific). Asterisks (*) refer to the protein bands. [file MPP-26-e70065-s007.docx]

**
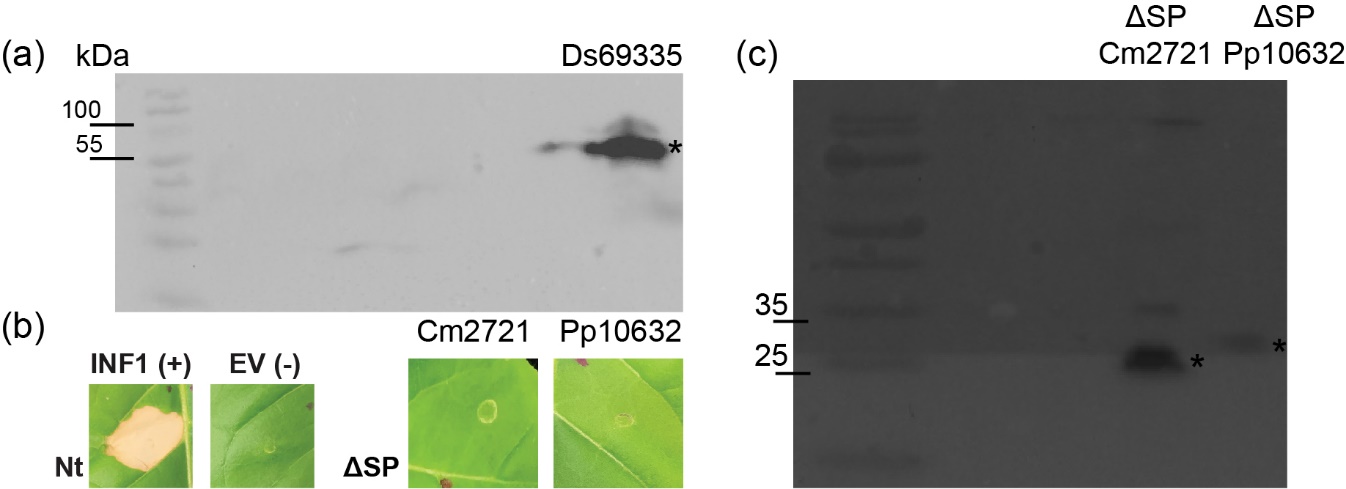
**

**Figure S1.** (a) Western blot detection of Ds69335 protein from *Dothistroma septosporum* that did not trigger chlorosis or cell death when expressed in *Nicotiana benthamiana*. (b) Proteins that previously triggered consistent cell death in non-host *Nicotiana tabacum* (Nt), Cm2721 and Pp10632, were expressed without a signal peptide (ΔSP) using an *Agrobacterium tumefaciens*-mediated transient expression assay to assess their ability to elicit cell death. INF1, *Phytophthora infestans* elicitin positive cell death control; EV, empty vector negative no-cell death control. Photos were taken 7 days after infiltration. (c) Western blot detection of Cm2721 and Pp10632 that lack the PR1α signal peptide (ΔSP). Anti-FLAG antibody was used for immuno-detection. Marker on the left of each membrane was the PageRuler™ Prestained Protein Ladder (ThermoScientific). Asterisks (*) refer to the protein bands.
