# Supplementary material for: Foliar Pine Pathogens From Different Kingdoms Share Defence‐Eliciting Effector Proteins
Source: Mol Plant Pathol. 2025 Mar 2;26(3):e70065. doi: 10.1111/mpp.70065 (PMC11872807; doi:10.1111/mpp.70065)
Supplement: Supplementary file 3 — Figure S3. Predicted protein tertiary structures of Ds69335 from Dothistroma septosporum, Cm8840 from Cyclaneusma minus and Pp7927 from Phytophthora pluvialis. Predicted structures of (a) Ds69335, (b) Cm8840 and (c) Pp7927. Protein structures were predicted with AlphaFold2, rendered in PyMol v. 2.5 (DeLano, 2002; Jumper et al., 2021; Mirdita et al., 2022) and coloured according to their AlphaFold2 pLDDT score: dark blue for regions predicted with high confidence, light blue and green for regions of low confidence and red for very low confidence regions. Disulphide bonds and/or cysteine residues are shown as yellow sticks. In Ds69335, cysteine residues at positions 150 and 230, and at positions 225 and 248, likely form disulphide bonds. The same is also likely for cysteine residues at positions 103 and 183 of Cm8840. (d) Characterised structure of Pry1 from Saccharomyces cerevisiae (Research Collaboratory for Structural Bioinformatics protein data bank [RCSB PBD] ID: 5jys) (Darwiche et al. 2016). [file MPP-26-e70065-s005.docx]

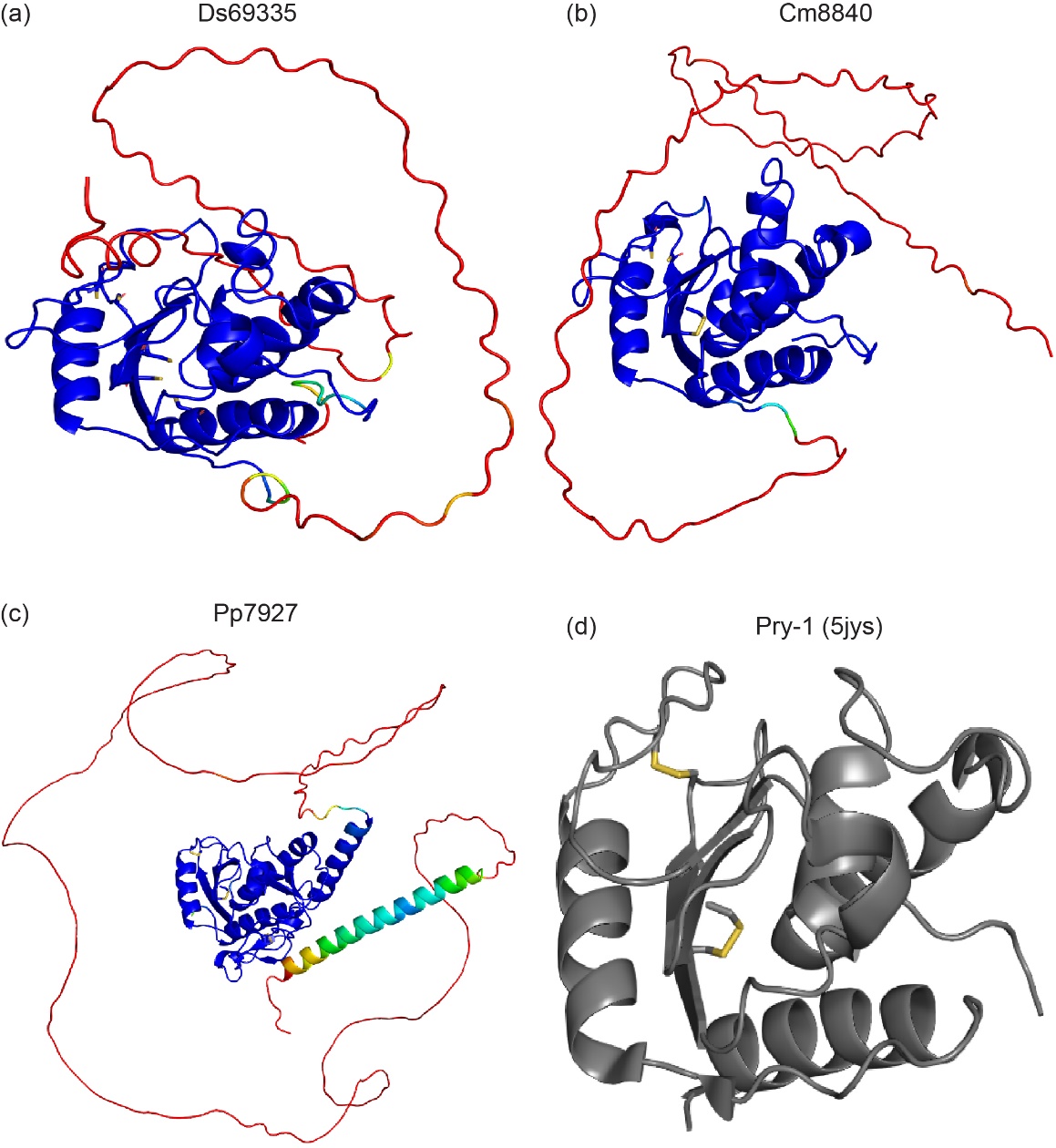


**Figure S3.** Predicted protein tertiary structures of Ds69335 from *Dothistroma septosporum*, Cm8840 from *Cyclaneusma minus* and Pp7927 from *Phytophthora pluvialis*. Predicted structures of (a) Ds69335, (b) Cm8840 and (c) Pp7927. Protein structures were predicted with AlphaFold2, rendered in PyMol v2.5 (DeLano, 2002; Jumper et al., 2021; Mirdita et al., 2022), and coloured according to their AlphaFold2 pLDDT score: dark blue for regions predicted with high confidence, light blue and green for regions of low confidence, and red for very low confidence regions. Disulphide bonds and/or cysteine residues are shown as yellow sticks. In Ds69335, cysteine residues at positions 150 and 230, and at positions 225 and 248, likely form disulphide bonds. The same is also likely for cysteine residues at positions 103 and 183 of Cm8840. (d) Characterized structure of Pry1 from *Saccharomyces cerevisiae* (Research Collaboratory for Structural Bioinformatics protein data bank [RCSB PBD] ID: 5jys) (Darwiche et al., 2016).

**References**

Darwiche, R., Kelleher, A., Hudspeth, E. M., Schneiter, R. and Asojo, O. A. (2016). Structural and functional characterization of the CAP domain of pathogen-related yeast 1 (Pry1) protein. *Scientific Reports, 6*, 28838.

DeLano, W. L. (2002). Pymol: an open-source molecular graphics tool. *CCP4 Newsletter on Protein Crystallography*, *40*, 82–92.

Jumper, J., Evans, R., Pritzel, A., et al. (2021). Highly accurate protein structure prediction with AlphaFold. *Nature, 596*, 583–589.

Mirdita, M., Schütze, K., Moriwaki, Y., Heo, L., Ovchinnikov, S. & Steinegger, M. (2022). ColabFold: making protein folding accessible to all. *Nature Methods, 19*, 679–682.
