# Supplementary material for: Foliar Pine Pathogens From Different Kingdoms Share Defence‐Eliciting Effector Proteins
Source: Mol Plant Pathol. 2025 Mar 2;26(3):e70065. doi: 10.1111/mpp.70065 (PMC11872807; doi:10.1111/mpp.70065)
Supplement: Supplementary file 4 — Figure S4. Dothistroma septosporum CRISPR/Cas9 Ds69335 disruption and confirmation by PCR and Southern hybridization. (a) Schematic diagram showing the disruption of Ds69335 and insertion of the nptII geneticin resistance gene cassette (PtrpC‐nptII‐TtrpC) through homologous recombination, using donor DNA (dDNA) as template. The dDNA was constructed with two flanks (5′ and 3′) from Ds69335, starting 3 bp from the double‐strand break (shown as the vertical black line crossing the gene), with the nptII cassette in the middle. Positions of primers are illustrated by grey flags with the primer name above. Also shown are the restriction enzyme sites and probe binding site (red line) used for Southern hybridization and the fragment sizes expected from the disruption of Ds69335 by insertion of the nptII cassette. (b) PCR amplicons generated with primers MT91 and MT92, which bind to the start and stop regions of the coding sequence, respectively. Ds69335 mutants should have a product of 3.6 kb, while the wild‐type (WT) should have one of 0.89 kb. (c) PCR amplicons generated with primers MT93 and MT94, which bind either side of the target genomic region. Ds69335 mutants should have a product of 5.4 kb, and the WT fungus 2.6 kb. Relevant size labels are shown on the right of each gel. (d) Southern hybridization of StuI and SmaI‐digested gDNA from D. septosporum WT fungus and six candidate Ds69335 mutants (1, 2, 4, 7, 36, 40) with SmaI and five with StuI (2, 4, 7, 36, 40) using a digoxigenin (DIG)‐11‐dUTP‐labelled probe binding to the 3′ flank region of Ds69335 that was present in the dDNA. Expected fragment sizes are marked with a red asterisk for Ds69335 mutants and black for WT fungus. Each of the five Ds69335 mutants were sampled from independent transformation plates. (e) PCR screening of putative Ds69335‐T2 complementation strains with primers MT91 and MT92, which bind to the start and stop of the coding sequence, respectively. Complementation strains should have a produc [file MPP-26-e70065-s017.docx]

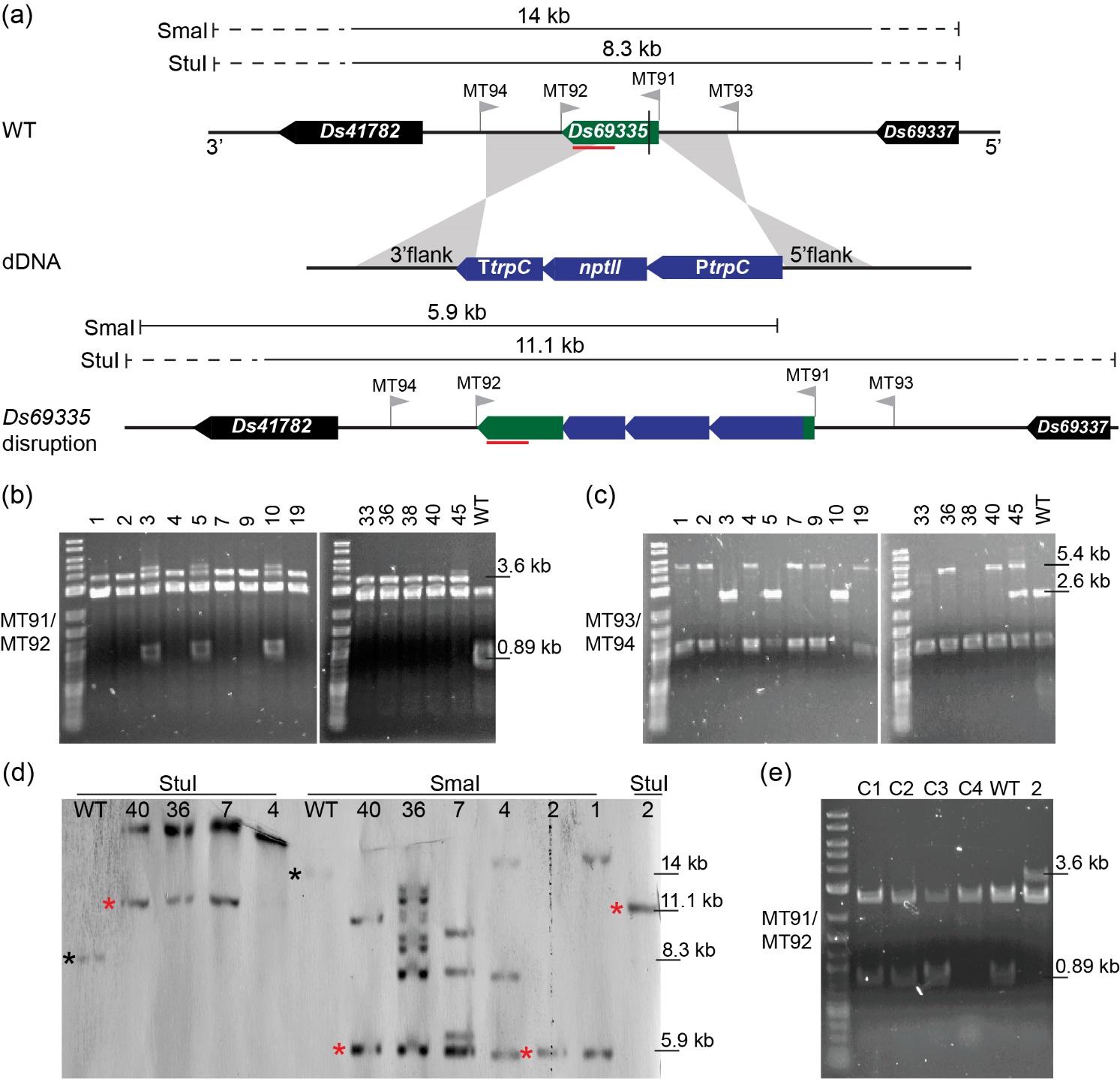


**Figure S4.** *Dothistroma septosporum* CRISPR/Cas9 *Ds69335* disruption and confirmation by PCR and Southern hybridization. (a) Schematic diagram showing the disruption of *Ds69335* and insertion of the *nptII* geneticin resistance gene cassette (P*trpC*-*nptII*-T*trpC*) through homologous recombination, using donor DNA (dDNA) as template. The dDNA was constructed with two flanks (5’ and 3’) from *Ds69335*, starting 3 bp from the double-strand break (shown as the vertical black line crossing the gene), with the *nptII* cassette in the middle. Positions of primers are illustrated by grey flags with the primer name above. Also shown are the restriction enzyme sites and probe binding site (red line) used for Southern hybridization and the fragment sizes expected from the disruption of *Ds69335* by insertion of the *nptII* cassette. (b) PCR amplicons generated with primers MT91 and MT92, which bind to the start and stop regions of the coding sequence, respectively. *Ds69335* mutants should have a product of 3.6 kb, while the wild-type (WT) should have one of 0.89 kb. (c) PCR amplicons generated with primers MT93 and MT94, which bind either side of the target genomic region. *Ds69335* mutants should have a product of 5.4 kb, and the WT fungus 2.6 kb. Relevant size labels are shown on the right of each gel. (d) Southern hybridization of StuI and SmaI-digested gDNA from *D. septosporum* WT fungus and six candidate *Ds69335* mutants (1, 2, 4, 7, 36, 40) with SmaI and five with StuI (2, 4, 7, 36, 40) using a digoxigenin (DIG)-11-dUTP-labeled probe binding to the 3’ flank region of *Ds69335* that was present in the dDNA. Expected fragment sizes are marked with a red asterisk for *Ds69335* mutants and black for WT fungus. Each of the five *Ds69335* mutants were sampled from independent transformation plates. (e) PCR screening of putative *Ds69335*-T2 complementation strains with primers MT91 and MT92, which bind to the start and stop of the coding sequence, respectively. Complementation strains should have a product of 0.89 kb, the same as the WT fungus, and a 3.6 kb from the *nptII* cassette insertion. Lanes C1-C4 show the complementation strains, lane 2 shows the *Ds69335*-T2 mutant.
