# Supplementary material for: Foliar Pine Pathogens From Different Kingdoms Share Defence‐Eliciting Effector Proteins
Source: Mol Plant Pathol. 2025 Mar 2;26(3):e70065. doi: 10.1111/mpp.70065 (PMC11872807; doi:10.1111/mpp.70065)
Supplement: Supplementary file 5 — Figure S5. Expression of Nicotiana benthamiana defence‐related genes in response to production of Ds131885, Cm2721 and Pp10632. Expression of defence‐related marker genes (a, d and g) NbPR1a and NbPR2, salicylic acid‐dependent immunity; (b, e and h) NbPR4 and NbLOX, jasmonic acid‐dependent immunity; (c, f and i) ERF1, ethylene‐dependent immunity in N. benthamiana. Ds131885, Cm2721, Pp10632and empty pICH86988 vector (EV) were expressed in N. benthamiana using an Agrobacterium tumefaciens ‐mediated transient expression assay and leaves were sampled after 48 h. Transcript levels were normalised to the reference gene NbActin and compared to the level of the control (set as 1). Means and standard errors were calculated from at least three biological replicates. *p < 0.05, **p < 0.01, ***p < 0.001. [file MPP-26-e70065-s001.docx]

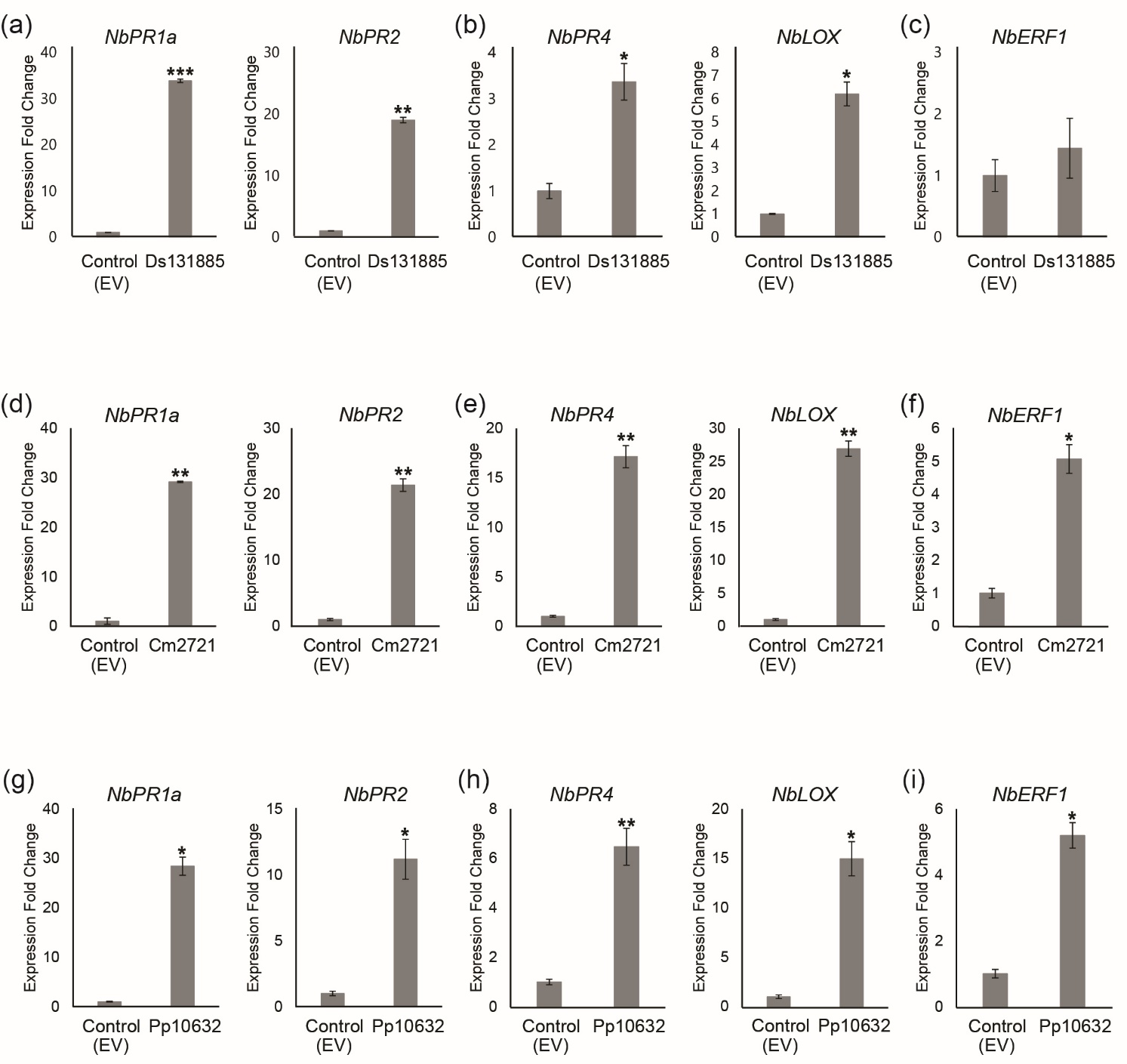


**Figure S5.** Expression of *Nicotiana benthamiana* defence-related genes in response to production of Ds131885, Cm2721 and Pp10632. Expression of defence-related marker genes (a, d and g) *NbPR1a* and *NbPR2*, salicylic acid-dependent immunity; (b, e and h) *NbPR4* and *NbLOX*, jasmonic acid-dependent immunity; (c, f and i) *ERF1*, ethylene-dependent immunity in *N. benthamiana*. Ds131885, Cm2721, Pp10632and empty pICH86988 vector (EV) were expressed in *N. benthamiana* using an *Agrobacterium tumefaciens*-mediated transient expression assay and leaves were sampled after 48 h. Transcript levels were normalized to the reference gene *NbActin* and compared to the level of the control (set as 1). Means and standard errors were calculated from at least three biological replicates. *, *P*<0.05; **, *P*<0.01; ***, *P*<0.001.
