# Supplementary material for: Foliar Pine Pathogens From Different Kingdoms Share Defence‐Eliciting Effector Proteins
Source: Mol Plant Pathol. 2025 Mar 2;26(3):e70065. doi: 10.1111/mpp.70065 (PMC11872807; doi:10.1111/mpp.70065)
Supplement: Supplementary file 6 — Figure S6. Dothistroma septosporum CRISPR/Cas9 Ds131885 disruption and confirmation by PCR and Southern hybridization. (a) Schematic diagram showing the disruption of Ds131885 and insertion of the nptII geneticin gene cassette (PtrpC‐nptII‐TtrpC) through homologous recombination, using donor DNA (dDNA) as template. The dDNA was constructed with two flanks (5′ and 3′) from Ds131885, starting 3 bp from the double‐strand break (shown as the vertical black line crossing the gene), with the nptII cassette in the middle. Positions of primers are illustrated by grey flags with the primer name above. Also shown are the restriction enzyme sites and probe binding site (red line) used for Southern hybridization and the fragment sizes expected from the disruption of Ds131885 by insertion of the nptII cassette. (b) PCR amplicons generated with primers MT99 and MT100, which bind to the start and stop regions of the coding sequence, respectively. Ds131885 mutants should have a product of 3.3 kb, and the wild‐type (WT) fungus 0.54 kb. (c) PCR amplicons generated with primers MT101 and MT102, which bind on either side of the target genomic region. Ds131885 mutants should have a product of 4.7 kb, and the WT fungus 1.9 kb. Relevant size labels are shown on the right of each gel. (d) Southern hybridization of EcoRI and NdeI‐digested gDNA from D. septosporum WT fungus and six candidate Ds131885 mutants, 2, 9, 13, 25, 46 and 47, using a digoxigenin (DIG)‐11‐dUTP‐labelled probe binding to the 3′ flank region of Ds131885 that was present in the dDNA. Expected fragment sizes are marked with a red asterisk for Ds131885 mutants and black for WT fungus. Each of the six Ds131885 mutants were sampled from independent transformation plates. (e) PCR screening of putative Ds131885‐T9 complementation strains with primers MT99 and MT100, which bind to the start and stop of the coding sequence, respectively. Complementation strains should have a product of 0.54 kb, the same as the WT fungus, and 3.3 [file MPP-26-e70065-s012.docx]

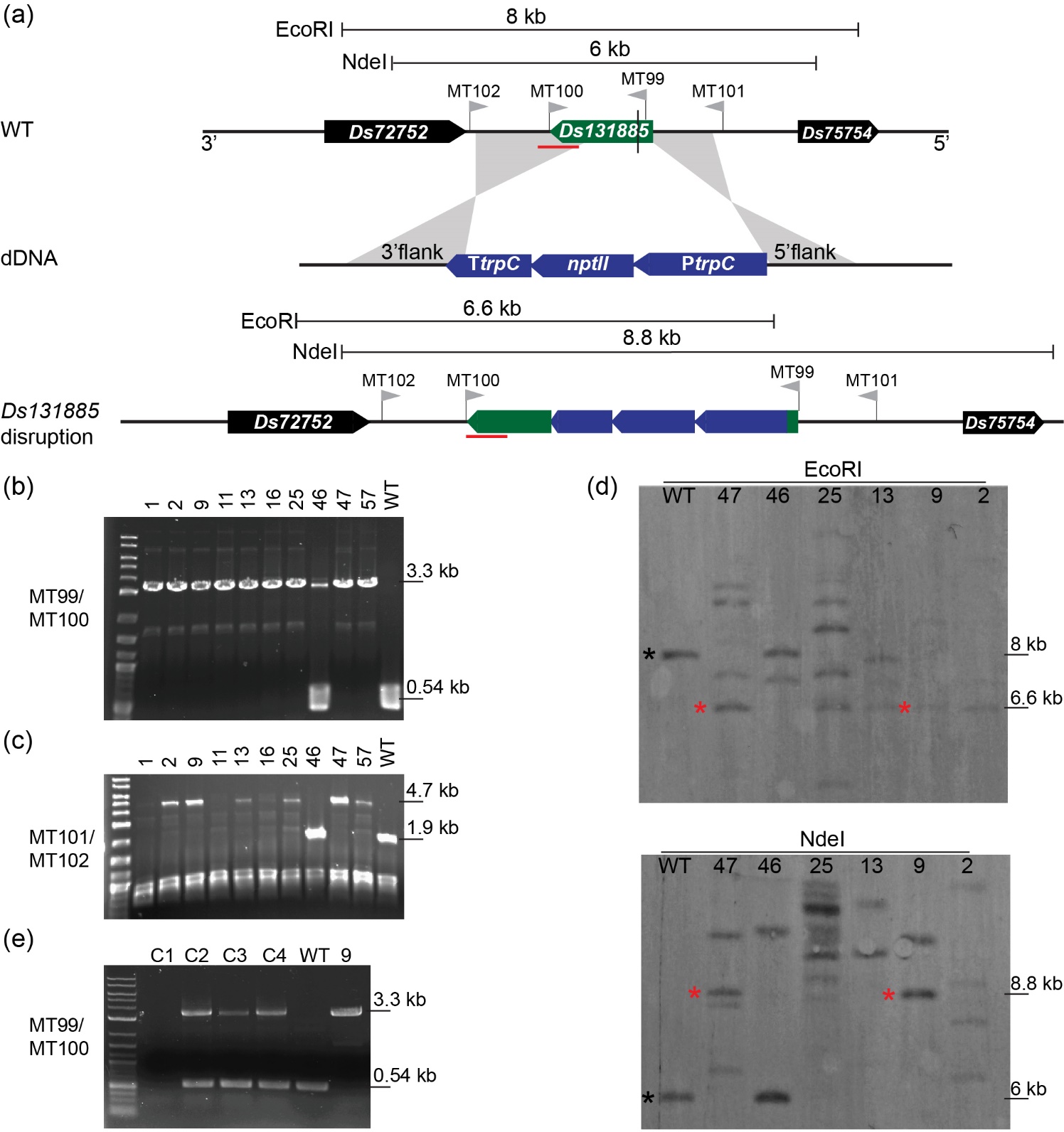


**Figure S6.** *Dothistroma septosporum* CRISPR/Cas9 *Ds131885* disruption and confirmation by PCR and Southern hybridization. (a) Schematic diagram showing the disruption of *Ds131885* and insertion of the *nptII* geneticin gene cassette (P*trpC*-*nptII*-T*trpC*) through homologous recombination, using donor DNA (dDNA) as template. The dDNA was constructed with two flanks (5’ and 3’) from *Ds131885*, starting 3 bp from the double-strand break (shown as the vertical black line crossing the gene), with the *nptII* cassette in the middle. Positions of primers are illustrated by grey flags with the primer name above. Also shown are the restriction enzyme sites and probe binding site (red line) used for Southern hybridization and the fragment sizes expected from the disruption of *Ds131885* by insertion of the *nptII* cassette. (b) PCR amplicons generated with primers MT99 and MT100, which bind to the start and stop regions of the coding sequence, respectively. *Ds131885* mutants should have a product of 3.3 kb, and the wild-type (WT) fungus 0.54 kb. (c) PCR amplicons generated with primers MT101 and MT102, which bind either side of the target genomic region. *Ds131885* mutants should have a product of 4.7 kb, and the WT fungus 1.9 kb. Relevant size labels are shown on the right of each gel. (d) Southern hybridization of EcoRI and NdeI-digested gDNA from *D. septosporum* WT fungus and six candidate *Ds131885* mutants, 2, 9, 13, 25, 46 and 47, using a digoxigenin (DIG)-11-dUTP-labeled probe binding to the 3’ flank region of *Ds131885* that was present in the dDNA. Expected fragment sizes are marked with a red asterisk for *Ds131885* mutants and black for WT fungus. Each of the six *Ds131885* mutants were sampled from independent transformation plates. (e) PCR screening of putative *Ds131885*-T9 complementation strains with primers MT99 and MT100, which bind to the start and stop of the coding sequence, respectively. Complementation strains should have a product of 0.54 kb, the same as the WT fungus, and a 3.3 kb from the *nptII* cassette insertion. Lanes C1-C4 show the complementation strains, lane 9 shows the *Ds131885*-T9 mutant.
