# Supplementary material for: Foliar Pine Pathogens From Different Kingdoms Share Defence‐Eliciting Effector Proteins
Source: Mol Plant Pathol. 2025 Mar 2;26(3):e70065. doi: 10.1111/mpp.70065 (PMC11872807; doi:10.1111/mpp.70065)
Supplement: Supplementary file 7 — Figure S7. Features of β‐trefoil proteins from Dothistroma septosporum, Cyclaneusma minus and Phytophthora pluvialis. (a) Proteins Cm2492 from C. minus and Pp12323 from P. pluvialis were expressed in Nicotiana benthamiana (top) and Nicotiana tabacum (lower) using an Agrobacterium tumefaciens ‐mediated transient expression assay (ATTA) to assess their ability to elicit chlorosis or cell death. Representative images are shown (n = 12–24 infiltration zones), from at least three independent experiments. INF1, Phytophthora infestans elicitin positive cell death control; EV, empty vector negative no‐cell death control. Photographs were taken 7 days after infiltration. (b) Proteins Ds74283 and DsEcp32‐1 from D. septosporum were expressed in wild‐type (WT) N. benthamiana and a N. benthamiana SOBIR1 deletion mutant (ΔSOBIR1) using an ATTA to assess their ability to elicit chlorosis or cell death. Representative images are shown (n = 12–24 infiltration zones), from at least three independent experiments. Positive (pos.) cell death control not requiring SOBIR1: Stemphylium lycopersici TW65_01570; negative (neg.) control requiring SOBIR1 for cell death: Avr9B/Cf‐9B. Photographs were taken 7 days after infiltration. (c) Predicted structure of Ds69113; AlphaFold2 predicted a tertiary structure with a pLDDT score of 90.7 and a predicted TM‐score of 0.88. (d) Alignment of predicted structures of D. septosporum β‐trefoil proteins Ds69113 (blue), Ds71487 (black), Ds74283 (grey) and DsEcp32‐1 (purple). The conserved disulphide bond is shown as red sticks. [file MPP-26-e70065-s013.docx]

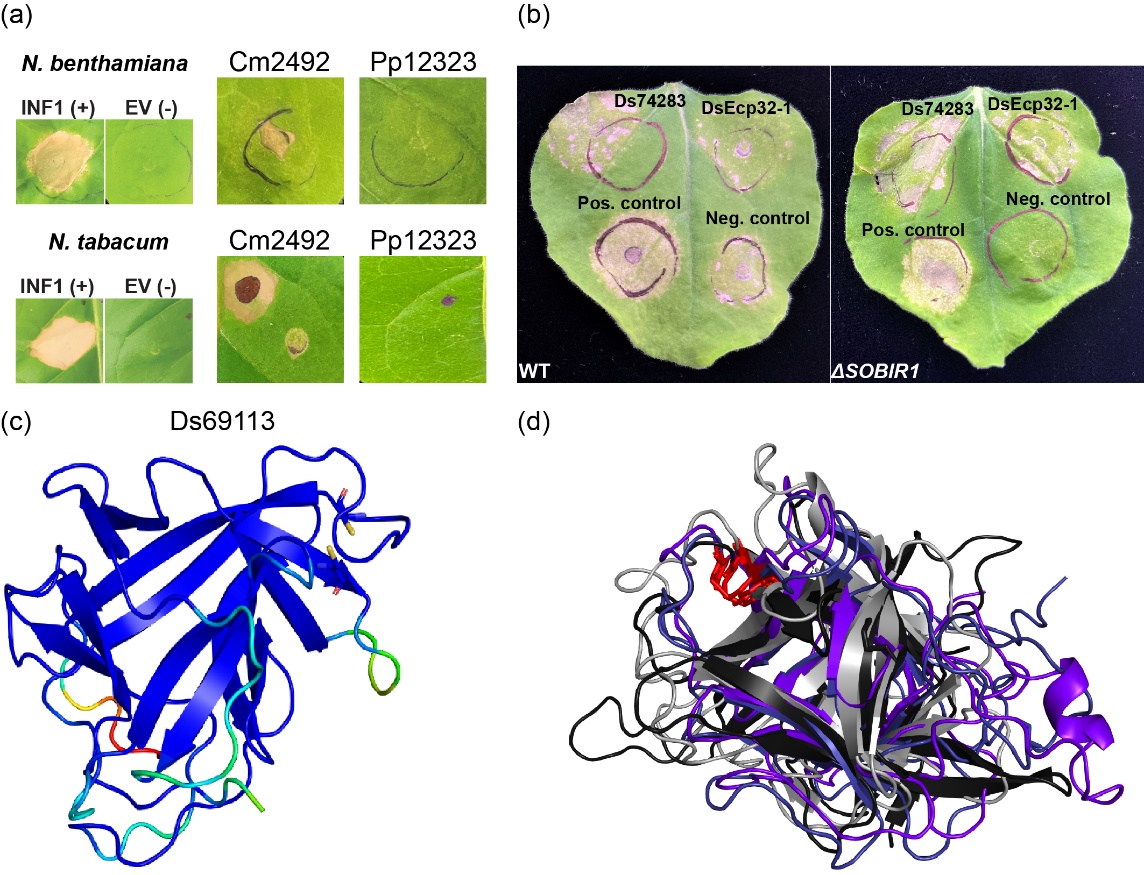


**Figure S7.** Features of β-trefoil proteins from *Dothistroma septosporum*, *Cyclaneusma minus* and *Phytophthora pluvialis*. (a) Proteins Cm2492 from *C. minus* and Pp12323 from *P. pluvialis* were expressed in *Nicotiana benthamiana* (top) and *Nicotiana tabacum* (lower) using an *Agrobacterium tumefaciens*-mediated transient expression assay (ATTA) to assess their ability to elicit chlorosis or cell death. Representative images are shown (n = 12–24 infiltration zones), from at least three independent experiments. INF1, *Phytophthora infestans* elicitin positive cell death control; EV, empty vector negative no-cell death control. Photos were taken 7 days after infiltration. (b) Proteins Ds74283 and DsEcp32-1 from *D. septosporum* were expressed in wild-type (WT) *N. benthamiana* and a *N. benthamiana* SOBIR1 deletion mutant (*ΔSOBIR1*) using an ATTA to assess their ability to elicit chlorosis or cell death. Representative images are shown (n = 12–24 infiltration zones), from at least three independent experiments. Positive (pos.) cell death control not requiring SOBIR1: *Stemphylium lycopersici* TW65_01570; negative (neg.) control requiring SOBIR1 for cell death: Avr9B/Cf-9B. Photos were taken 7 days after infiltration. (c) Predicted structure of Ds69113; AlphaFold2 predicted a tertiary structure with a pLDDT score of 90.7 and a predicted TM-score of 0.88. (d) Alignment of predicted structures of *D. septosporum* β-trefoil proteins Ds69113 (blue), Ds71487 (black), Ds74283 (grey) and DsEcp32-1 (purple). The conserved disulphide bond is shown as red sticks.
