# Supplementary material for: Foliar Pine Pathogens From Different Kingdoms Share Defence‐Eliciting Effector Proteins
Source: Mol Plant Pathol. 2025 Mar 2;26(3):e70065. doi: 10.1111/mpp.70065 (PMC11872807; doi:10.1111/mpp.70065)
Supplement: Supplementary file 8 — Figure S8. Dothistroma septosporum candidate effector Ds74283 triggers the expression of plant defence response genes in Nicotiana benthamiana. Expression of defence‐related marker genes (a) NbPR1a and NbPR2, salicylic acid (SA)‐dependent immunity; (b) NbPR4 and NbLOX, jasmonic acid (JA)‐dependent immunity; (c) ERF1, ethylene‐dependent immunity in N. benthamiana. Ds74283 and empty pICH86988 vector (EV) were expressed in N. benthamiana using an Agrobacterium tumefaciens ‐mediated transient expression assay and leaves were sampled after 24 h. Transcript levels were normalised to the reference gene NbActin and compared to the level of the control (set as 1). Means and standard errors were calculated from at least three biological replicates. *p < 0.05, **p < 0.01. [file MPP-26-e70065-s009.docx]

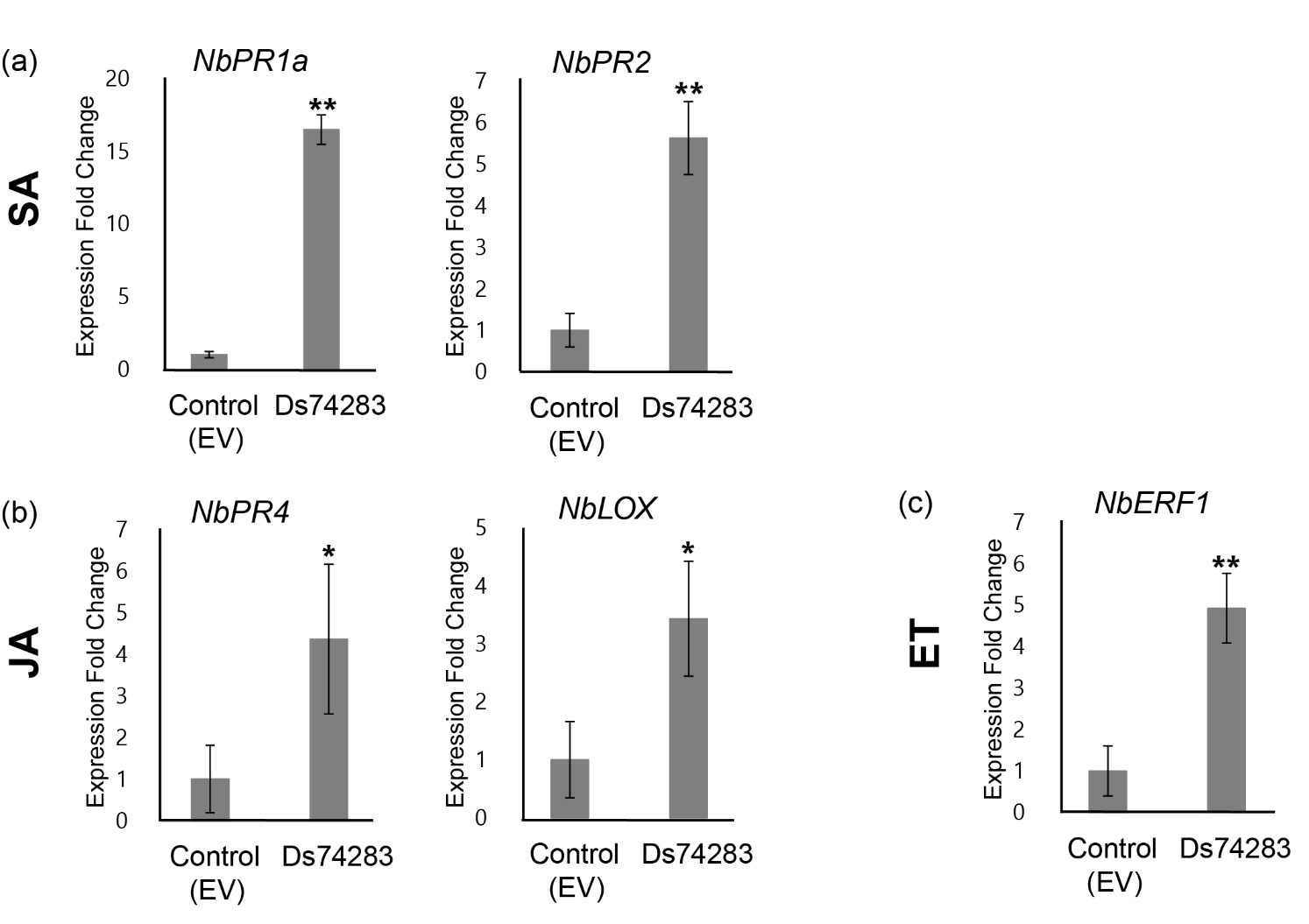


**Figure S8.** *Dothistroma septosporum* candidate effector Ds74283 triggers the expression of plant defence response genes in *Nicotiana benthamiana*. Expression of defence-related marker genes (a) *NbPR1a* and *NbPR2*, salicylic acid (SA)-dependent immunity; (b) *NbPR4* and *NbLOX*, jasmonic acid (JA)- dependent immunity; (c) *ERF1*, ethylene-dependent immunity in *N. benthamiana*. Ds74283 and empty pICH86988 vector (EV) were expressed in *N. benthamiana* using an *Agrobacterium tumefaciens*-mediated transient expression assay and leaves were sampled after 24 h. Transcript levels were normalized to the reference gene *NbActin* and compared to the level of the control (set as 1). Means and standard errors were calculated from at least three biological replicates. *, *P*<0.05; **, *P*<0.01.
