# Supplementary material for: Foliar Pine Pathogens From Different Kingdoms Share Defence‐Eliciting Effector Proteins
Source: Mol Plant Pathol. 2025 Mar 2;26(3):e70065. doi: 10.1111/mpp.70065 (PMC11872807; doi:10.1111/mpp.70065)
Supplement: Supplementary file 10 — File S1. Virulence assays of Dothistroma septosporum transformants in Pinus radiata . [file MPP-26-e70065-s004.docx]

**File S1.** Virulence assays of *Dothistroma septosporum* transformants in *Pinus radiata*. Further details of the virulence assays are described and discussed here.

**Methods details**

Candidate effector gene disruption and complementation strains of *D. septosporum*, as well as the WT fungus, were inoculated onto *P. radiata* seedlings. Two sets of experiments were performed at two separate times (Set A and Set B), and each set had its own WT control. This was done because not all strains produced enough spores in time for Set A. Each set had seedlings inoculated with the WT fungus, so transformants could be compared to the WT fungus of the respective time they were inoculated. Table 1 shows how many replicate pines were inoculated for each strain, and the concentration and volume of spores used, as well as which strains were inoculated in Set A and Set B.

**Table 1.** Details of the *Dothistroma septosporum* candidate effector gene disruption and complementation strain virulence assay experiment in *Pinus radiata*.

| ***CE* gene** | **Strain name^b^** | **Concentration (spores/mL)^c^** | **Volume (mL)^d^** | **Pine seedlings replicates** |
| --- | --- | --- | --- | --- |
| **Set A** | | | | |
|  | WT | 1.6 x 10^6^ | 65 | 3 |
| *Ds69335* | *Ds69335* T2-D | 1.6 x 10^6^ | 65 | 4 |
|  | *Ds69335* T40-D | 1.6 x 10^6^ | 62 | 4 |
|  | *Ds69335* T2-Co | 1.6 x 10^6^ | 55 | 4 |
| *Ds74283* | *Ds74283* T3-D | 1.6 x 10^6^ | 60 | 4 |
|  | *Ds74283* T56-D | 1.6 x 10^6^ | 50 | 4 |
|  | *Ds74283* T3-Co | 1.6 x 10^6^ | 65 | 4 |
| *Ds131885* | *Ds131885* T9-Co | 1.6 x 10^6^ | 65 | 4 |
| **Set B** | | | | |
|  | WT | 6 x 10^5^ | 65 | 3 |
| *Ds131885* | *Ds131885* T9-D (L) | 7.5 x 10^5^ | 35 | 3 |
|  | *Ds131885* T9-D (M) | 1 x 10^6^ | 50 | 3 |
|  | *Ds131885* T47-D | 1 x 10^6^ | 50 | 3 |

^a^ Joint Genome Institute (JGI) protein ID corresponding to the *Dothistroma septosporum* NZE10 (Ds) candidate effector (*CE*) gene of interest that was disrupted.

^b^ *D. septosporum* strains with a targeted candidate effector gene disruption are indicated by “D”; complementation strains are indicated by “Co”. WT: wild-type. Low (L) and moderate (M) concentration of spores used for inoculation of *Pinus radiata* seedlings.

^c^ Spore concentration in spores per millilitre (mL).

^d^ Total volume of spore solution sprayed in all replicates for each stain in mL. Note that not all sprayed spores will have landed on the fine pine needles, and it was therefore not possible to control the specific number of spores inoculated.

After the pines were sprayed, they were left to air-dry for 20 min. Each plant was then covered with a clean plastic sleeve (petri dish bag) to maintain very high needle wetness. Plants were placed in growth chambers (one replicate seedling inoculated with each strain per chamber), that consisted of a plastic container and a plastic frame covered with clear plastic using methods described by Kabir et al (2013). A ventilation opening was cut at the top that allowed for it to be opened or closed. Seedlings were placed on top of tip boxes to elevate the pots above the water level in the chamber. After four days, the plastic sleeve covering each plant was removed. Inside each chamber, two custom-made water misters/foggers were then placed in order to keep high needle wetness. Water was also sprayed through the ventilation opening at the top of the chamber to apply additional wetness once or twice a day, as necessary.

For the first two weeks for Set A of inoculated seedlings, and the first one week for set B of inoculated seedlings, the experiment was conducted in a growth room with artificial light (12 h light/12 h dark photoperiod with 180 μ mol m-2s-1) at 22°C. After that, seedlings were transferred to a 22°C growth room, with approximately 80% humidity and under natural light (15 to 750 μ mol m-2s-1) for an additional eight weeks. The experiments were conducted for a total of ten weeks for both sets of inoculated seedlings. The transfer from one set of conditions to another was because the growth room with natural light was not available at the start of either of the experiments.

All analyses were done by comparing the *D. septosporum* disruption strain either to the WT fungus in the same set in which they were inoculated, or with its corresponding complementation strain, regarding the number of DNB lesions and fungal biomass estimations.

After ten weeks, all needles were harvested and counted, and the percentages of needles with DNB lesions were calculated. The percentages of needles with lesions caused by each disruption and complementation strain were compared to those caused by the corresponding WT fungus, and disruptants were also compared to their respective complements (Table 2).

**Results**

The results suggested that gene disruption had no effect on the number of lesions when compared with the WT, except for *Ds131885* T47-D, which caused fewer lesions than the WT fungus, even though this strain was inoculated at a higher spore concentration than the Set B WT (Table 1). Also, disruption strains did not show any differences when compared to the respective complementation strains. There was high variability between replicate seedlings inoculated with the same strain, for example with *Ds69335* T2-Co, *Ds74283* T56-D and *Ds131885* T9-D (M).

**Table 2.** Percentage of needles with Dothistroma needle blight (DNB) lesion symptoms in response to infection with candidate effector gene disruption and complementation strains of *Dothistroma septosporum*.

| ***CE* gene^a^** | **Strain name^b^** | **Percentage (%) of needles with DNB lesions^c^** | ***P***  **(T-test) - WT^d^** | ***P***  **(T-test) - Co^e^** |
| --- | --- | --- | --- | --- |
| **Set A** | | | | |
|  | WT | 13.8±3.22 | - | - |
| *Ds69335* | *Ds69335* T2-D | 12.4±3.29 | 0.65 | 0.33 |
|  | *Ds69335* T40-D | 16.2±7.76 | 0.68 | 0.67 |
|  | *Ds69335* T2-Co | 19.9±12.02 | 0.50 | - |
| *Ds74283* | *Ds74283* T3-D | 22.8±5.25 | 0.08 | 0.81 |
|  | *Ds74283* T56-D | 23.9±9.92 | 0.21 | 0.75 |
|  | *Ds74283* T3-Co | 21.4±7.93 | 0.24 | - |
| *Ds131885* | *Ds131885* T9-Co | 13.3±3.83 | 0.89 | - |
| **Set B** | | | | |
|  | WT | 24.9±3.53 | - | - |
| *Ds131885* | *Ds131885* T9-D (L) | 19.3±6.37 | 0.34 | 0.24 |
|  | *Ds131885* T9-D (M) | 26.6±10.21 | 0.83 | 0.10 |
|  | *Ds131885* T47-D | 7.9±7.9 | 0.003* | 0.10 |

^a^ Joint Genome Institute (JGI) protein ID corresponding to the *Dothistroma septosporum* NZE10 (*Ds*) candidate effector (*CE*) gene of interest that was disrupted.

^b^ *D. septosporum* strains with a targeted candidate effector gene disruption are indicated by “D”; complementation strains are indicated by “Co”. WT: wild-type. Low (L) and moderate (M) concentration of spores used for inoculation of *Pinus radiata* seedlings according to Table 1.

^c^ Mean (± standard deviation) of needles with DNB lesions compared with total number of needles in each inoculated seedling, shown as percentage (%). Full data shown in Tarallo (2022).

^d^ *P*>0.05, using Student’s *t*-test, indicates no significant difference between WT and each transformant. The asterisk * indicates significant difference.

^e^ *P*>0.05, using Student’s *t*-test, indicates no significant difference between the disruption strain and its respective complement.

To determine if the gene disruption had any effects on pathogen growth on *P. radiata*, fungal biomass in characteristic DNB lesions was estimated using a qPCR assay comparing the single-copy *DsPksA* gene from *D. septosporum* and the *CAD* gene from *P. radiata* (Table 3). Figure 1 shows a graphical representation of the values obtained for each inoculated seedling for all transformants (both disruption and complementation strains) and WT, in relative fungal biomass per dry-weight, and fungal biomass per number of lesions. The results highlight the variability between replicates for most of the samples. The results (Figure 1a) showed that in Set A all disruption strains differed in the amount of biomass normalized by dry-weight when compared with the WT fungus. However, when compared to their respective complementation strain, none of the disruption strains showed any differences in biomass. The amount of fungal biomass was also normalized by the number of lesions (Figure 1B). In this case, no significant differences in the amount of fungal biomass were observed between any of the samples. In Set B, *Ds131885* T9-D (L) and *Ds131885* T47-D did not show any differences when compared to the WT fungus in ng/mg dry-weight, although *Ds131885* T9-D (M) appeared to have a lower amount of fungal biomass than the WT. None of the disrupted transformants were different from their complements regarding the amount of biomass per dry-weight or number of lesions.

**Table 3.** Copy of Table 1 from main manuscript **-** Relative quantification of *Dothistroma septosporum* candidate effector gene disruption and complementation strain biomass in *Pinus radiata*.

| ***DsCE* gene^a^** | **Strain name^b^** | **Fungal DNA (rel quant) (ng/mg DW)^c^** | ***P* (T-test)**  **WT/Co^d^** |
| --- | --- | --- | --- |
| **Set A** |  |  |  |
|  | WT | 7.1 ± 0.6 | - |
| *Ds69335* | *Ds69335* T2-D | 3.6 ± 1.6 | 0.025* / 0.23 |
|  | *Ds69335* T40-D | 3.0 ± 2.7 | 0.078 / 0.58 |
|  | *Ds69335* T2-Co | 1.9 ± 1.5 | 0.006* / - |
| *Ds74283* | *Ds74283* T3-D | 2.4 ± 1.5 | 0.007* / 0.51 |
|  | *Ds74283* T56-D | 2.7 ± 1.0 | 0.003* / 0.24 |
|  | *Ds74283* T3-Co | 1.6 ± 1.0 | 0.001* / - |
| *Ds131885* | *Ds131885* T9-Co | 1.3 ± 1.9 | 0.008* / - |
| **Set B** |  |  |  |
|  | WT | 2.0 ± 0.5 | - |
| *Ds131885* | *Ds131885* T9-D (L) | 3.5 ± 1.6 | 0.30 / 0.24 |
|  | *Ds131885* T9-D (M) | 0.7 ± 0.2 | 0.03* / 0.65 |
|  | *Ds131885* T47-D | 3.3 ± 1.0 | 0.18 / 0.22 |

^a^ Joint Genome Institute (JGI) protein ID corresponding to the *Dothistroma septosporum* NZE10 (*Ds*) candidate effector (*CE*) gene of interest that was disrupted.

^b^ *D. septosporum* strains with a targeted gene disruption are indicated by “D”; complementation strains are indicated by “Co”. WT: wild-type. Low (L) and moderate (M) concentration of spores used for inoculation of *Pinus radiata* seedlings according to Table 1.

^c^ Fungal biomass (ng) relative to pine and normalised to dry-weight (DW) of infected needle tissue (mean ± SD), determined by the cycle threshold (Ct) amplification values from qPCR of *DsPksA* (fungal target) and *CAD* (pine reference), based on regression equations: y = -3.7597x + 26.877 (target) and y = -3.365x + 27.84 (reference). Full data shown in Tarallo (2022).

^d^ *P*-value, using Student’s *t*-test, between WT and each transformant (first value) and between the disruption strain and its respective complementation strain (second value). The asterisk * indicates significant difference (*P*<0.05).


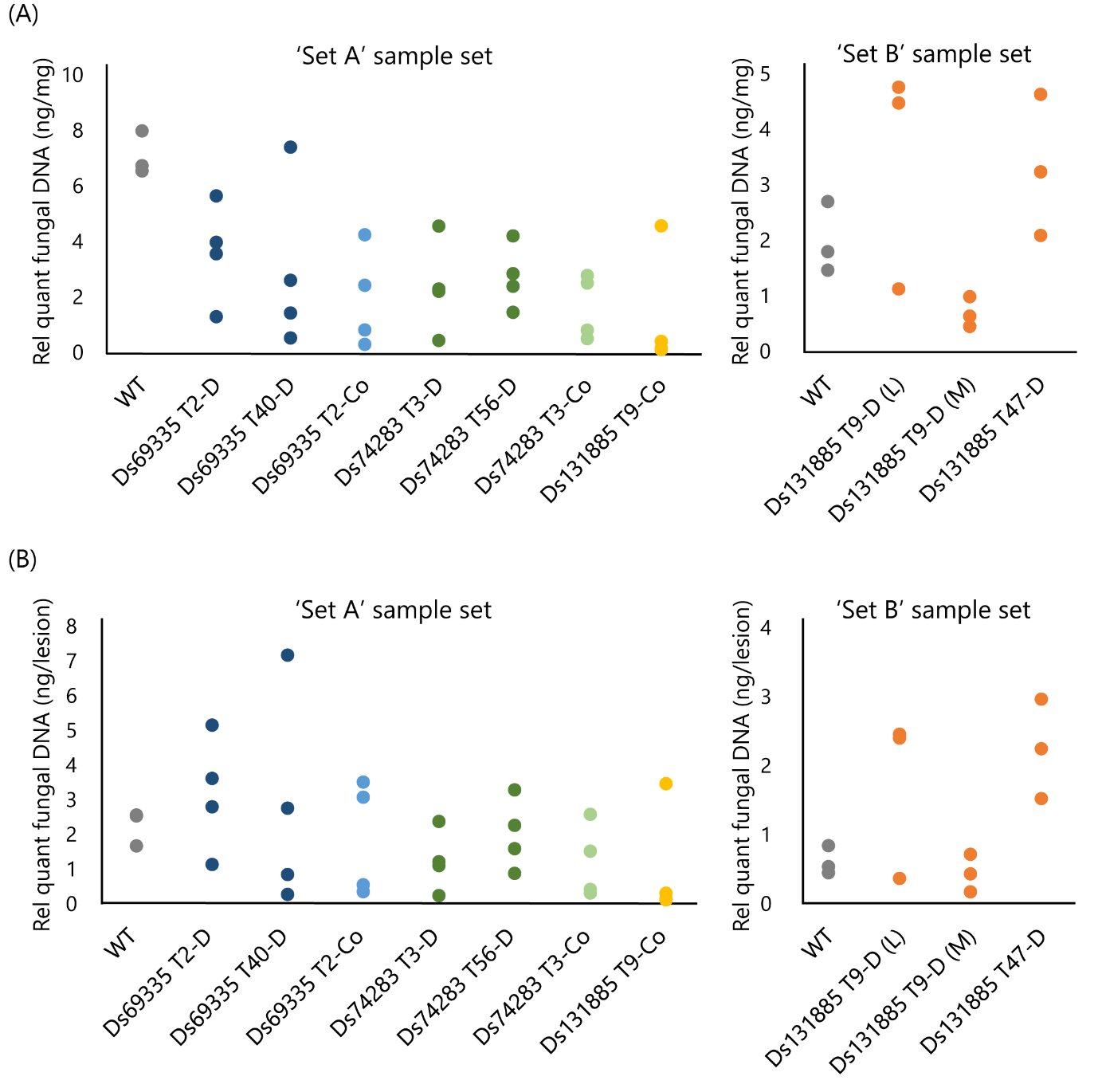


**Figure 1.** Relative quantification of *Dothistroma septosporum* candidate effector gene disruption and complementation strain biomass following *Pinus radiata* infection. Fungal biomass (ng) in characteristic Dothistroma needle blight lesions was estimated using a qPCR assay comparing the *DsPksA* gene from *D. septosporum* and the *CAD* gene from *P. radiata* for each *D. septosporum* disruption or complementation strain and wild-type (WT) fungus. Values were normalized by dry-weight (mg) (A) and number of lesions (B). ‘Set A’ and ‘Set B’ refers to the experiment set up according to Table 1. D: disrupted *D. septosporum* strain; Co: complementation strain of *D. septosporum*; L: low spore concentration; M: moderate spore concentration compared to those of other strains. Full data shown in Tarallo (2022).

**Discussion**

The virulence assay showed that none of the disrupted genes were required for pathogenicity in *D. septosporum*, since all transformants were still able to cause characteristic DNB lesions. As for virulence, all Set A strains (with disruption of *Ds69335* or *Ds74283*) showed a decrease in fungal biomass in the infected needle tissue (Figure 1). However, when comparing the amount of fungal biomass between all the complementation strains and WT, no clear conclusion could be drawn, as the complementation strains also had significantly less fungal biomass. This was observed despite all inoculations in Set A having the same starting concentration and similar volumes of spores, and the seedlings being maintained in the same experimental conditions. These results suggest that the complementation strains were not restored in virulence. It is important to note that non-clonal *P. radiata* seedlings were used in this experiment due to the unavailability of clonal seedlings; this could have contributed to variability between replicates, as differences in host genotype could lead to different responses to *D. septosporum* infection.

Complementation is carried out to ensure that the phenotype of a transformant with a targeted gene mutation is due only to that specific mutation and not ectopic effects. It is possible that the *Ds69335*- and *Ds74283*-disrupted strains (Set A) actually did have reduced fungal biomass and therefore reduced virulence compared to the WT, but that factors associated with the complementation process prevented restoration of virulence. When transforming the mutant to generate a complementation strain, the functional copy of the gene is often inserted at a random ectopic place in the genome. The position of the CE genes in the genome could be important for their expression and some might be under chromatin regulation, which compromised their expression. Future experiments should include a larger number of complementation strains for testing; here only one from each gene was used due to space limitations in the virulence assay.

Another possible explanation for the lack of complementation is that off-target effects occurred in the complementation strains due to Cas9 expression. Constitutive expression of Cas9 is known to increase the number of off-target effects and DNA damage (Cho et al., 2014; Fu et al., 2013; Pattanayak et al., 2013). The Cas9 gene may have integrated into the *D. septosporum* genome during the transformation process, enabling the endonuclease to target and cleave the inserted functional copy of the target gene in the three complementation strains. Also, mismatches in the complementarity between the sgRNA and target gene sequences are tolerated and might have led to the Cas9 cleaving other regions of the genome (Pattanayak et al., 2013), generating other types of off-target events. All transformants had additional hybridising fragments in the Southern analysis that did not match with sizes expected for additional gene copies or partial digestion (probes were designed to specifically hybridize with each candidate gene (Supp. Figures S4 and S6). This suggests that parts of the Cas9HygAMAccdB plasmid or pAN-7 might have integrated into the genome, concomitant with the CE genes being disrupted. In the case of Ds74283 and Ds131885, disrupted transformants were kept on selective hygromycin medium after protoplast regeneration. The expression vector Cas9HygAMAccdB is only lost after the removal of the hygromycin selection, which means the Cas9 was retained for longer, increasing the chance of integration into the genome. This was also shown to be case for some *V. inaequalis* CRISPR/Cas9 transformants, in which the Cas9HygAMAccdB plasmid had integrated into their genomes (Rocafort et al., 2022).

For future experiments, the PAM site in complementing genes should be mutated to prevent any unwanted Cas9 activity. Other forms of Cas9 delivery could be exploited, such as transient delivery of Cas9, in the form of a ribonucleoprotein which has a limited half-life, directly into the cell, in order to limit off-target effects and possible integration into the genome of the transformant (Yip, 2020). The integration of the Cas9 gene, if accompanied with integration of the specific sgRNA in the genomes of the disruption strains, would explain why all complementation strains have similar low fungal biomass amount when compared to the disruption strains, as the additional gene copy in each of these strains might also have been mutated.

Aside from problems with the complementation strains, there were also limitations in the virulence assay due to variation in inoculum concentrations and the availability of growth rooms (Tarallo, 2022). Another limitation concerned the sampling time of the experiment. Here, the experiment was conducted for ten weeks after inoculation. After ten weeks, DNB lesion symptoms were advanced with abundant sporulating lesions and some needle death, which corresponds to the Late (necrotrophic) infection stage of *D. septosporum* according to previous studies (Bradshaw et al., 2016; Kabir et al., 2015). By focusing on the single 10 weeks post-inoculation time point it was not possible to determine if the rate of infection differed between the strains. Instead, the single late sampling point might have masked any real differences in the virulence capacity of the disrupted strains that may have been evident at earlier stages.

While the quantification of fungal biomass showed a lower trend for both disruption and complementation transformants when compared to the WT, the percentages of infected needles were not significantly different between any of these transformants and the WT fungus, or between the gene-disrupted strains and their respective complementation strains. Measuring the lesion sizes produced by the transformants in this study might also provide additional information rather than just counting the total number of lesions produced.

We understand that, whilst additional work is needed, it is important to note that pine virulence assays take a long time; once the trees are grown and ready for use (1–2 years depending on if seedlings or clones are used), it takes at least four months from obtaining sufficient inoculum through to completing fungal biomass analysis of the infected seedlings, so unfortunately, we were not able to repeat the experiments shown in this study. However, despite the technical challenges with these experiments, particularly the problems related to the complementation strains, we have shown a tentative biomass reduction compared to the WT as an indication that these effectors (Ds69335 and Ds74283) might be involved in virulence and warrant further investigation.

**References**

Bradshaw, R. E., Guo, Y., Sim, A. D., *et al*. (2016). Genome-wide gene expression dynamics of the fungal pathogen *Dothistroma septosporum* throughout its infection cycle of the gymnosperm host *Pinus radiata*. *Molecular Plant Pathology, 17*, 210–224.

Cho, S. W., Kim, S., Kim, Y., et al. (2014). Analysis of off-target effects of CRISPR/Cas derived RNA-guided endonucleases and nickases. *Genome Research, 24*, 132–141.

Fu, Y., Foden, J. A., Khayter, C., et al. (2013). High-frequency off-target mutagenesis induced by CRISPR-Cas nucleases in human cells. *Nature Biotechnology, 31*, 822–826.

Kabir, M. S., Ganley, R. J. and Bradshaw, R. E. (2013). An improved artificial pathogenicity assay for Dothistroma needle blight on *Pinus radiata*. *Australasian Plant Pathology, 42*, 503–510.

Kabir, M. S., Ganley, R. J. and Bradshaw, R. E. (2015). The hemibiotrophic lifestyle of the fungal pine pathogen *Dothistroma septosporum*. *Forest Pathology, 45*, 190–202.

Pattanayak, V., Lin, S., Guilinger, J. P., Ma, E., Doudna, J. A. & Liu, D. R. (2013). High-throughput profiling of off-target DNA cleavage reveals RNA-programmed Cas9 nuclease specificity. *Nature Biotechnology, 31*, 839–843.

Rocafort, M., Arshed, S., Hudson, D., et al. (2022). CRISPR-Cas9 gene editing and rapid detection of gene-edited mutants using high-resolution melting in the apple scab fungus, *Venturia inaequalis*. *Fungal Biology, 126*, 35–46.

Tarallo, M. (2022). *Identification and characterization of effector proteins from pine needle pathogens.* [PhD thesis]. Massey University, Manawatū, New Zealand.

Yip, B. H. (2020). Recent advances in CRISPR/Cas9 delivery strategies. *Biomolecules, 10*, 839.
